# *Taf1* knockout is lethal in embryonic male mice and heterozygous females show weight and movement disorders

**DOI:** 10.1242/dmm.050741

**Published:** 2024-07-10

**Authors:** Elisa M. Crombie, Andrea J. Korecki, Karen Cleverley, Bethany A. Adair, Thomas J. Cunningham, Weaverly Colleen Lee, Tess C. Lengyell, Cheryl Maduro, Victor Mo, Liam M. Slade, Ines Zouhair, Elizabeth M. C. Fisher, Elizabeth M. Simpson

**Affiliations:** ^1^ Department of Neuromuscular Diseases, UCL Institute of Neurology, University College London, London WC1N 3BG, UK; ^2^ Centre for Molecular Medicine and Therapeutics at BC Children's Hospital, University of British Columbia, Vancouver, BC V5Z 4H4, Canada; ^3^ Department of Medical Genetics, University of British Columbia, Vancouver V6T 1Z3, Canada; ^4^ MRC Harwell Institute, Harwell Campus, Oxfordshire OX11 0RD, UK

**Keywords:** TATA box-binding protein-associated factor 1, X-linked dystonia–parkinsonism, Male lethality, X-linked intellectual disability, X inactivation, Genetic mouse model, Transcription initiation complex

## Abstract

The TATA box-binding protein-associated factor 1 (TAF1) is a ubiquitously expressed protein and the largest subunit of the basal transcription factor TFIID, which plays a key role in initiation of RNA polymerase II-dependent transcription. *TAF1* missense variants in human males cause X-linked intellectual disability, a neurodevelopmental disorder, and *TAF1* is dysregulated in X-linked dystonia–parkinsonism, a neurodegenerative disorder. However, this field has lacked a genetic mouse model of TAF1 disease to explore its mechanism in mammals and treatments. Here, we generated and validated a conditional cre-lox allele and the first ubiquitous *Taf1* knockout mouse. We discovered that *Taf1* deletion in male mice was embryonically lethal, which may explain why no null variants have been identified in humans. In the brains of *Taf1* heterozygous female mice, no differences were found in gross structure, overall expression and protein localisation, suggesting extreme skewed X inactivation towards the non-mutant chromosome. Nevertheless, these female mice exhibited a significant increase in weight, weight with age, and reduced movement, suggesting that a small subset of neurons was negatively impacted by *Taf1* loss. Finally, this new mouse model may be a future platform for the development of TAF1 disease therapeutics.

## Teaser

**Summary:** Variants in *TAF1* cause X-linked intellectual disability and X-linked dystonia–parkinsonism. A novel conditional cre-lox allele and ubiquitous knockout mouse model for *Taf1* can be used to study TAF1 disease.

## INTRODUCTION

The TATA box-binding protein-associated factor 1 (TAF1) is a ubiquitously expressed protein that plays a key role in initiation of RNA polymerase II-dependent transcription. *TAF1* is best known for forming a protein complex with other TAFs to form the basal transcription factor TFIID and to regulate the activation machinery for transcription initiation complex assembly (Bhuiyan and Timmers, 2019; Malik and Roeder, 2023). The *TAF1* gene is located on the X chromosome (Xq13.1); thus, in males, there is only one copy, and in females, there are two, one of which undergoes X inactivation to maintain the same dosage in both sexes (Deng et al., 2014).

*TAF1* missense variants in males (maternally inherited or acquired *de novo*) can cause the neurodevelopmental disorder X-linked syndromic intellectual developmental disorder-33 (MRXS33) also known as X-linked intellectual disability (XLID), which may present with craniofacial abnormalities and a variety of other clinical features including congenital heart disease (Gudmundsson et al., 2019; O'Rawe et al., 2015). *TAF1* missense variants in females have been reported with similar phenotypes as those in males (Giovenino et al., 2023; Gudmundsson et al., 2019). However, many female cases are asymptomatic and were identified due to their relationship with affected male family members (Gudmundsson et al., 2019). In both males and females, almost all *TAF1* variants were missense. Only two unrelated families carried gene duplications including *TAF1* (and surrounding genes at Xq13.1), resulting in a severe progressive neurodegenerative phenotype, but with few common clinical features to cases with missense variants (O'Rawe et al., 2015). Finally, just one splice site variant has been identified in an asymptomatic mother and her affected son (O'Rawe et al., 2015). Therefore, the genotype–phenotype correlation between *TAF1* variants and clinical outcomes is not straightforward, and there are very little data regarding the relationship between mutation and mRNA/protein expression of TAF1. Currently, there are no successful treatments for these TAF1 disorders.

In addition, *TAF1* is dysregulated in X-linked dystonia–parkinsonism (XDP), an adult-onset neurodegenerative disorder, affecting individuals with ancestry from the Philippines, due to the insertion of a ∼2.6 kb SINE-VNTR-Alu (SVA) retrotransposon into intron 32 of the gene (Bragg et al., 2017; Makino et al., 2007). The SVA retrotransposon is reported to alter the splicing of *TAF1* transcripts (Aneichyk et al., 2018; Bragg et al., 2017; Ito et al., 2016; Makino et al., 2007) and to reduce total TAF1 at the mRNA (Rakovic et al., 2018) and protein (Makino et al., 2007) levels. Brains from individuals with XDP have striatal atrophy and patients manifest late-onset motor impairment (Goto et al., 2005). In most male patients, XDP is characterised initially by dystonia (93.4% of cases) that generalises within 5-10 years from the age of onset (∼39.5 years in males), which eventually transitions into parkinsonism (Lee et al., 2001, 2011). Typically affected males die around 16 years after diagnosis (Rosales, 2010). A few female XDP cases have been reported at a ratio of 1:100 (females:males), with a mean age of onset of 46 years; these individuals present with variable dystonia–parkinsonism features (Lee et al., 2011). However, as with *TAF1* variants, many mild or asymptomatic female cases may go undetected without genetic testing outside of families with affected members (Evidente et al., 2002, 2004) and, thus, the true proportion of affected versus unaffected females with XDP is difficult to estimate. Currently, there are no interventions that have achieved success in the treatment of XDP (Bragg et al., 2019).

In both females carrying *TAF1* variants (Cheng et al., 2019; Hurst et al., 2018; O'Rawe et al., 2015) and those with XDP (Domingo et al., 2014), skewed X inactivation (a process whereby inactivation of one X chromosome is selected over the other) towards the non-mutant chromosome has been suggested to explain the lack of, or minimal, phenotypes in some individuals (Evidente et al., 2004). However, in some females, carrying a *TAF1* variants (Cheng et al., 2019; Giovenino et al., 2023) or the XDP allele (Domingo et al., 2014) results in disease features despite reports of skewed X inactivation; this indicates that the phenotype is not masked and it has even been suggested that the mutant allele is more highly expressed in such cases (Domingo et al., 2014).

Model organisms are essential to define the role of TAF1 in disease, and various knockout (KO) and knockdown mutations have been published. In yeast (*Saccharomyces cerevisiae*), temperature-sensitive deletion of different TAF1 domains indicated that ∼90% of the yeast genome requires TAF1 for transcription (Irvin and Pugh, 2006); others showed that nearly all mRNA is strongly dependent on TFIID function (Warfield et al., 2017). In worms (*Caenorhabditis elegans*), RNAi-mediated knockdown showed that TAF1 in the TFIID complex is important for embryonic transcription (Walker et al., 2004). Very few vertebrate models of TAF1 dysfunction exist. In zebrafish (*Danio rerio*), homozygous KO of *taf1* was embryonically lethal, showing neurodevelopmental defects (Gudmundsson et al., 2019). To deplete *taf1* gene expression in zebrafish embryos, a transient *in vivo* splice-blocking morpholino approach was taken (O'Rawe et al., 2015); both approaches reduced the area of the optic tectum (midbrain) indicating microencephaly, comparable to that in human patients with XLID (Gudmundsson et al., 2019; O'Rawe et al., 2015). Two rat models with postnatal knockdown of *Taf1* have been published. In the first model, intracerebral injection into postnatal day 3 rats with lentivirus vectors carrying CRISPR/Cas9 reagents was used to target exon 1, resulting in behavioural and neurodevelopmental defects (Janakiraman et al., 2019). In the second model, intrastriatal infusions into 3-week-old rats with adeno-associated viral vectors carrying microRNAs was used to target *Taf1* in a splice variant-selective manner (Cirnaru et al., 2021), resulting in similar motor deficits. These same targeting constructs were used with intracerebroventricular injection into the brains of newborn mice, and phenotypes were similar to, or more even pronounced than, those in the rat models (Cirnaru et al., 2021).

Although these various models have provided important insights into TAF1 function, the field has lacked a genetic mouse model of TAF1 disease to explore its mechanism and treatments. Despite the recognition of limitations in all models, mice are well recognised as the premier genetic model (Eppig et al., 2017) and conditional cre-lox alleles are currently the most powerful tools (Kaloff et al., 2017). Such a mouse model will allow us to assess the role of TAF1 from the very earliest developmental stages and throughout life. It will allow molecular investigation of *TAF1* transcription and protein expression, coupled with gross brain anatomy and behaviour studies to reveal disease mechanisms. Finally, a well-validated mouse model will be a critical platform for therapy development to address these serious developmental and neurodegenerative diseases.

## RESULTS

### Generation and sequence verification of *Taf1* conditional KO allele

**Fig. 1.**
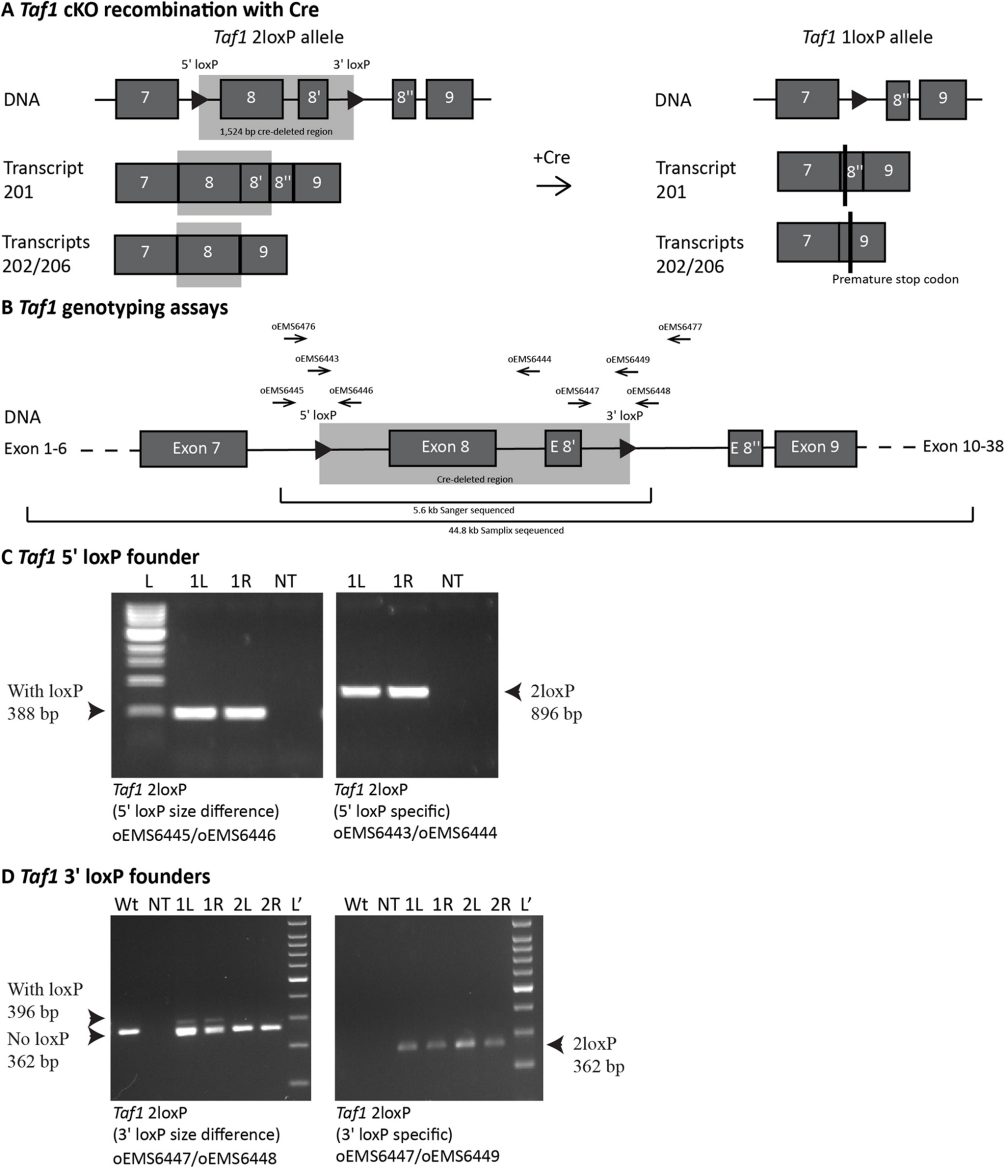
**Generation of the *Taf1* conditional knockout mouse strain.** (A,B) Schematics of the targeted region of the *Taf1* gene (not to scale). (A) The schematic shows the locations of the two loxP sites (triangles), 1524 bp cre-deleted region (grey box) and premature stop codons (black vertical lines). (B) The primers used in genotyping the locus are shown (horizontal arrows), and the Sanger- and Samplix-sequenced regions are indicated (horizontal brackets). (C,D) Genomic DNA amplification from founder mice was performed. Primer locations are indicated in B and sequences in Table S2. To assay for mosaicism, notches were studied from both ears (1L and 1R indicate samples from the left and right ears of mouse number 1). (C) Genomic DNA amplification from the male 5′ loxP founder. The amplicon from the primers oEMS6445 and oEMS6446 indicated the presence of the 5′ loxP by a size shift [354 (not present) to 388 bp]. The primer oEMS6443 specifically binds the 3′ loxP and its use with oEMS6444 indicated the presence of the 3ʹ loxP. L, 1 kb DNA ladder. (D) Female (mouse number 1) and male (mouse number 2) founders also carried the 3′ loxP, with the female founding strain C57BL/6J-*Taf1^em2ems^*. The amplicon from the primers oEMS6447 and oEMS6448 indicated the presence of the 3′ loxP by a size shift [362 to 396 bp (faint band indicates mosaics)]. The primer oEMS6449 specifically binds the 3′ loxP and its use with oEMS6447 indicated the presence of the 3ʹ loxP in both founders. Lʹ, 100 bp DNA ladder; NT, no template.

Fig. 1A shows a schematic of the *Taf1* conditional KO (cKO) allele, which follows the design recommendations of the International Knockout Mouse Consortium (Wefers et al., 2011). The loxP sites flank exon 8, which is present in all known *Taf1* transcripts, and exons 8 and 8ʹ in transcript 201 (Ensembl transcripts: ENSMUST00000101341.9, ENSMUST00000118878.8 and ENSMUST00000149274.9). The 1524-bp deletion is designed to create a frameshift that will result in downstream stop codons. The loxP sites were added in two steps. First, CRISPR strategies designed to simultaneously add both loxP sites were tested in embryonic stem cells (ESCs). The optimal strategy (Table S1) was microinjected into C57BL/6J (B6) zygotes *ex vivo*. The resulting mice were genotyped with primers shown in Fig. 1B and Table S2. This resulted in one male founder, which passed the new allele to offspring, but with only the 5′ loxP site present (Fig. 1C). This 5′ loxP-carrying strain was then expanded and used for a second round of CRISPR to introduce the 3′ loxP site. For this second insertion, another CRISPR strategy was developed and optimised in ESCs. The optimal strategy (Table S1) was delivered using improved genome editing via oviductal nucleic acid delivery (i-GONAD) electroporation into B6-*Taf1* 5′ loxP late zygotes *in vivo*. This resulted in three founders: one female and two males. The female and one male each passed the new allele to offspring (Fig. 1D), which resulted in strains named C57BL/6J-*Taf1^em1Ems^* and C57BL/6J-*Taf1^em2Ems^* (called *Taf1^em1Ems^* and *Taf1^em2Ems^* hereon for brevity), respectively. Both strains were initially studied to ensure reproducibility.

A region of ∼5.6 kb encompassing ∼0.8 kb upstream of the 5′ loxP site to ∼1.5 bp downstream of the 3′ loxP site was Sanger sequenced from a hemizygous male ear notch DNA sample from the *Taf1^em1Ems^* and *Taf1^em2Ems^* strains (Fig. 1B). This revealed that *Taf1^em1Ems^* had two single base pair deletions within a 3 bp intronic region, whereas *Taf1^em2Ems^* showed complete integrity of the expected *Taf1* sequence plus the two loxP sites. Although we did not observe any phenotypic impact of this unplanned mutation, going forward, all studies focused on *Taf1^em2Ems^*. DNA prepared from the brain of another *Taf1^em2Ems^* hemizygous male with two loxP sites was sent for Samplix indirect sequence capture analysis (Table S3) (Blondal et al., 2021) and showed the expected DNA sequence in a ∼44.8 kb stretch of the targeted locus (chromosome X: 101,522,350-101,567,153), including ∼34.8 kb with >100× coverage (chromosome X: 101,524,973-101,559,808), with >1000× coverage over the region of interest.

### Ubiquitous KO of *Taf1* resulted in 1loxP heterozygous females but no 1loxP hemizygous males

For brevity, the unrecombined conditional *Taf1* allele is referred to as 2loxP, as two loxP sites are present in the genome, and the recombined *Taf1* allele is referred to as 1loxP, as after deletion, there is only one loxP site remaining.

The introduction of loxP sites alone, without cre deletion, can change the transcription of a gene, causing an unplanned phenotype. Thus, we undertook characterisation of unrecombined 2loxP *Taf1^em1Ems^* and *Taf1^em2Ems^* strains. The 2loxP heterozygous females and 2loxP hemizygous males bred with the typical fertility and fecundity of B6 wild-type (Wt) mice. Both maternal and paternal transmission to offspring was as expected. At least three mice of each genotype were aged to at least 1 year and no abnormal phenotype was observed. For the *Taf1^em2Ems^* strain, 2loxP homozygous females were generated and bred to 2loxP hemizygous males. The 2loxP homozygous females also had typical fertility and fecundity, and transmission to offspring was as expected. During all these breeding experiments, no cage-level phenotypes were observed in the 2loxP mice. Thus, we conclude that the introduction of the two loxP sites into the introns of *Taf1* was without detrimental consequence.

**Fig. 2.**
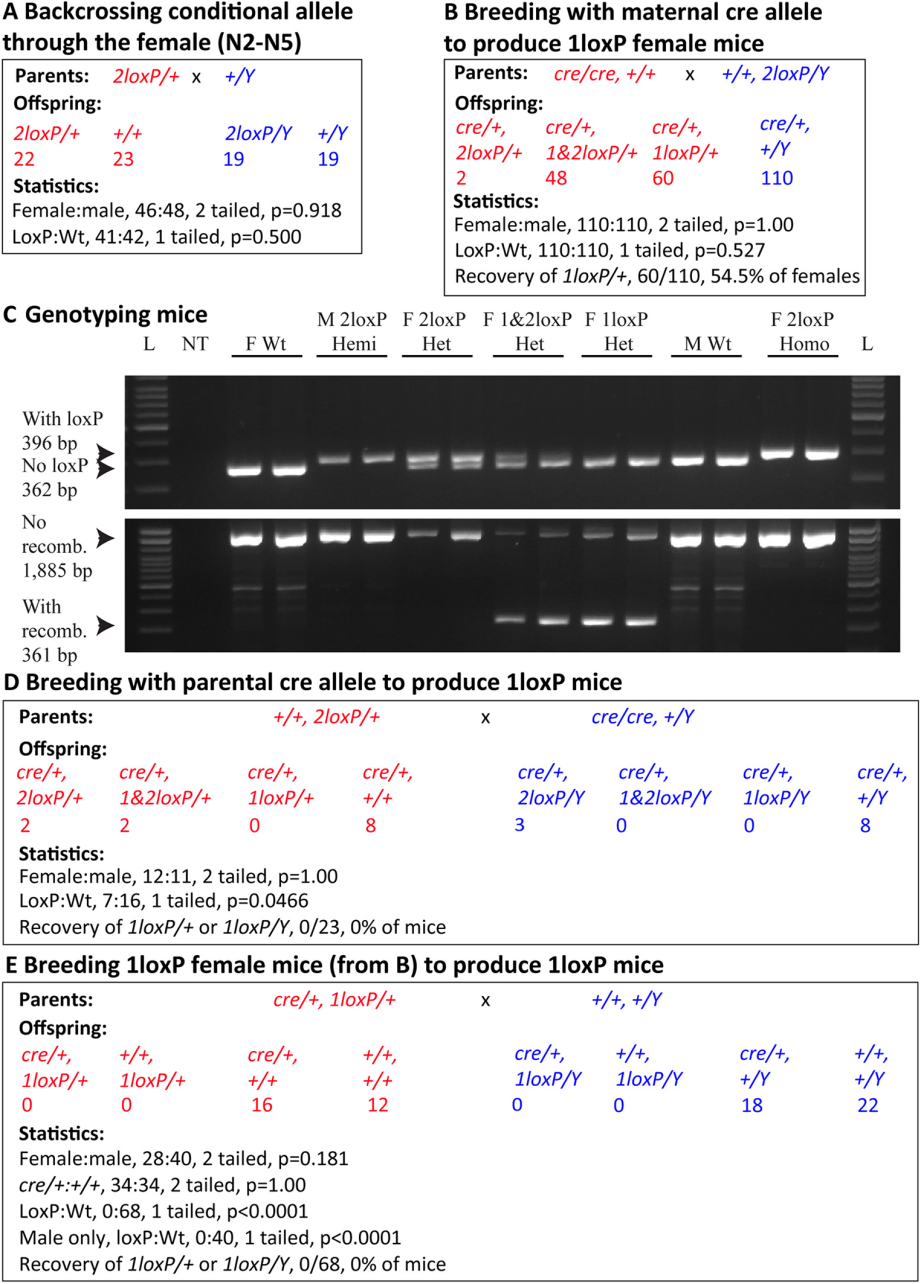
**Breeding produced *Taf1* 1loxP heterozygous females, but no 1loxP hemizygous males were born.** (A) The conditional knockout (cKO) 2loxP allele was generated and then maintained on the C57BL/6J background by backcrossing the X-linked allele typically through the female. Over all generations N2-N5, statistical analysis showed that the female:male ratio was not significantly different from that expected and, regardless of sex, the loxP:Wt ratio was also not significantly different from that expected. Note that not all mice sexed were genotyped. (B) Breeding homozygous EIIa-cre females to 2loxP hemizygous males resulted in unrecombined 2loxP, mosaic 1&2loxP and fully recombined 1loxP heterozygous females. As expected, only wild-type (Wt) males were produced. Statistical analysis showed an equal female:male ratio. Regardless of sex, equal loxP:Wt mice were born. 54.5% of females carried a fully recombined 1loxP. (C) Genomic DNA amplification assays resulted in unique banding patterns for all genotypes generated in A and B. Additionally, data are presented for 2loxP homozygous females (‘F’), which were generated by breeding 2loxP heterozygous females to 2loxP hemizygous males (‘M’). Primer locations are indicated in Fig. 1B and sequences in Table S2. L, 100 bp DNA ladder; NT, no template. (D) Breeding 2loxP heterozygous females to homozygous EIIa-cre males resulted in unrecombined 2 loxP and mosaic 1&2loxP, but no fully recombined 1loxP heterozygous females. This cross also resulted in unrecombined 2loxP, but no mosaic 1&2loxP, nor fully recombined 1loxP hemizygous males. As expected, the cross resulted in Wt females and Wt males. Statistical analysis showed that the female:male ratio was not significantly different from that expected. However, regardless of sex, the loxP:Wt ratio was significantly different from that expected, as 0% of mice carried only a recombined 1loxP. (E) Breeding 1loxP heterozygous females from B to Wt males resulted in no 1loxP mice of either sex being born. All mice were genotyped at birth. Statistical analysis showed that the female:male ratio was not significantly different from that expected, nor was the *cre*:Wt ratio. However, regardless of sex, or considering males only, the loxP:Wt ratio was highly significantly different from that expected, as 0% of mice carried a recombined 1loxP. Statistical analyses used the two-tailed binomial test for sex ratios and one-tailed binomial test for loxP:Wt as only loss of loxP animals was anticipated. Red, females; blue, males.

Although the strains were generated on the B6 background, we also backcrossed the mice to B6 to remove any unlinked mutations that might have been caused by the CRISPR-based methodology (Fig. 2A). For this we chose to breed the 2loxP allele through the female as, with an X-linked gene, this strategy produced both 2loxP heterozygous female and 2loxP hemizygous male mice. Backcrossing continued to N5 with no impact on expected inheritance, nor on fecundity or fertility. No other cage-level phenotypes were observed. Mice for molecular and histological characterisation were from the N4 and N5 backcrosses.

To mimic the human disease situation, in which a pathogenic variant is typically inherited or occurs very early in development, we bred the *Taf1^em2Ems^* 2loxP strain to the ubiquitously expressing B6.FVB-Tg(EIIa-cre)C5379Lmgd/J (hereafter EIIa-cre) (Lakso et al., 1996). EIIa-cre mice carry the *cre* transgene under the control of the adenovirus EIIa promoter that targets expression to the early mouse embryo, and is useful for widespread recombination of loxP sites across all tissues, including in the germ line. We performed the cross both ways, bringing the EIIa-cre transgene in from the maternal (Fig. 2B) or paternal (Fig. 2D) parent, as others have shown that the parental source of cre can affect recombination efficiency (Luo et al., 2020). We found in both cases that the loxP sites were able to recombine, deleting 1524 bp including exon 8 (or exons 8 and 8′) of the *Taf1* gene, but maternal transmission was more effective than paternal transmission. Maternal (Fig. 2B) transmission of *cre* generated both partially recombined mosaic females (*1&2loxP/+*) and completely recombined 1loxP heterozygous females. However, because of the location of *Taf1* on the X chromosome, 1loxP hemizygous males were not produced. Fig. 2C shows representative examples of genomic DNA amplification results for all mice, including partially recombined and completely recombined 1loxP heterozygous females. Paternal (Fig. 2D) transmission of *cre* should have generated both recombined 1loxP heterozygous female offspring and 1loxP hemizygous male offspring, but neither were recovered, despite the presence of partially recombined females (*1&2loxP/+*). Thus, we bred the first-generation 1loxP heterozygous females as shown in Fig. 2B to Wt males (Fig. 2E). Importantly, none passed a 2loxP chromosome to offspring, which could have happened if the females were mosaics. Despite scoring pups on the day of birth, no 1loxP heterozygous females nor 1loxP hemizygous males were recovered, which was highly significantly different from that expected. The lack of 1loxP heterozygous females may be due to the mouse-specific phenomenon of paternal X inactivation in extraembryonic tissues (in this cross the Wt X xhromosome was paternal), instead of the random pattern in humans (Huynh and Lee, 2005). Thus, we statistically considered the lack of 1loxP hemizygous males alone, which was also highly significantly different from that expected (loxP:Wt, 0:40; one-tailed binomial test, *P*<0.0001). From this, we concluded that in mice, dependence on the KO *Taf1* allele, which is designed to be protein null, is incompatible with postnatal life.

### *Taf1* 1loxP hemizygous male embryos survived to blastocyst stage and implanted

**Fig. 3.**
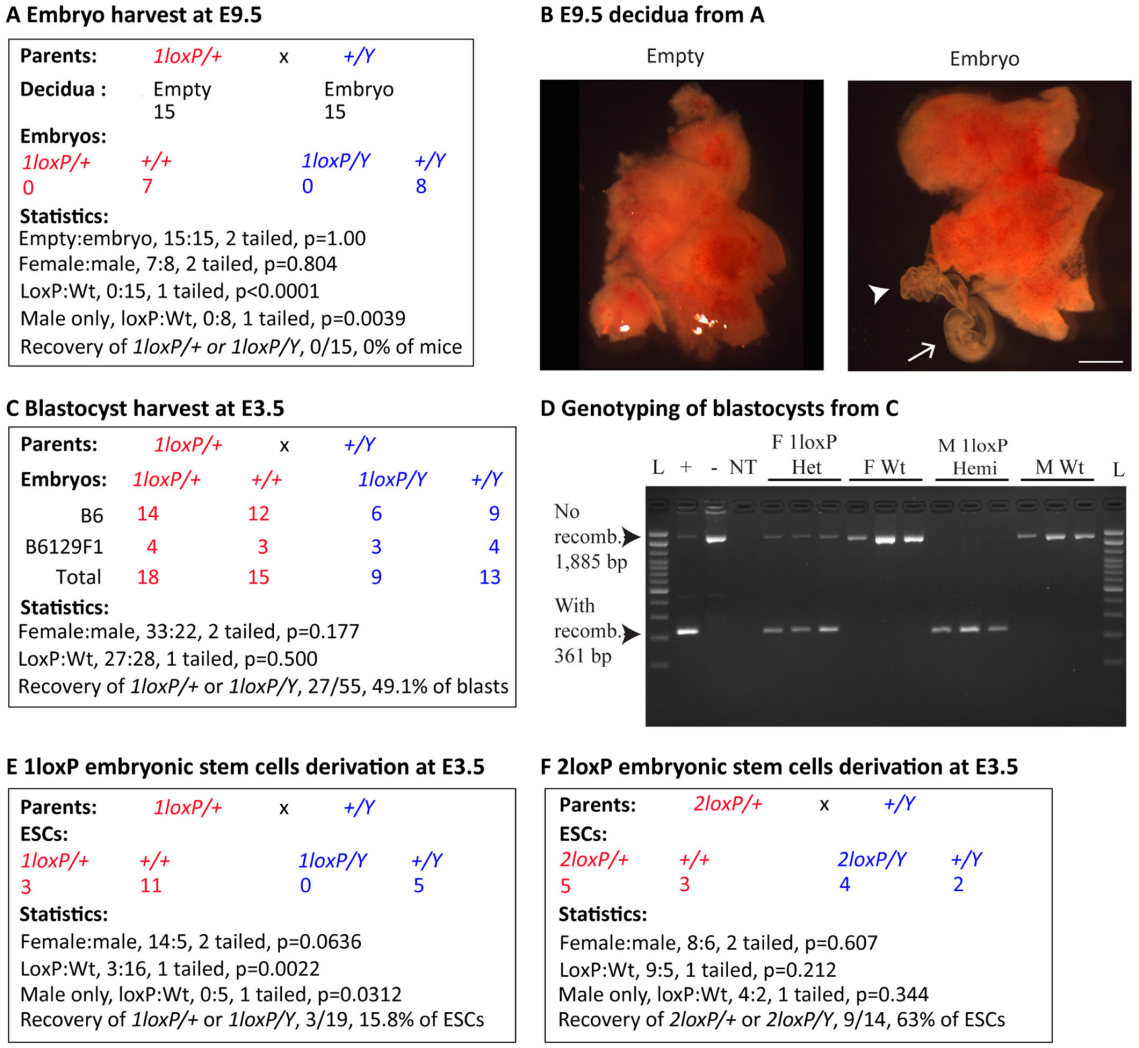
***Taf1* 1loxP hemizygous male embryos developed to blastocysts but resulted in no embryonic stem cells.** (A-F) Timed matings between 1loxP heterozygous females from Fig. 2B and Wt males (A-E) or between 2loxP heterozygous females from Fig. 2A and Wt males (F). (A,B) Harvest was at embryonic day (E) 9.5. (A) Dissection of decidua resulted in 15 empty decidua and 15 containing embryos within yolk sacs. Statistical analysis showed that the empty:embryo ratio was not significantly different from that expected assuming that empty decidua were induced by subsequently lost 1loxP embryos. Genomic DNA amplification of embryo-derived tissues resulted in detection of no 1loxP embryos of either sex. Statistical analysis showed that the female:male ratio was not significantly different from that expected. However, regardless of sex, or considering males only, the loxP:Wt ratio was highly significantly different from expected, as 0% of embryos carried a recombined 1loxP. (B) Representative images of empty and embryo-containing decidua from A. Arrowhead, yolk sac; arrow, embryo. Scale bar: 2 mm. (C-F) Harvest was at E3.5. (C) Blastocysts were harvested from both the B6 and B6129F1 mice. Surprisingly, genomic DNA amplification detected 1loxP embryos of both sexes from both B6 and B6129F1 mice. Statistical analysis of the total blastocysts showed that both the female:male and loxP:Wt ratios were not significantly different from those expected, and 49.1% of blastocysts carried a recombined 1loxP. (D) Representative genomic DNA amplification results for the four genotypes recovered as blastocysts in C. Primer locations are indicated in Fig. 1B and sequences in Table S2. L, 100 bp ladder; +, positive control ear notch from a 1loxP heterozygous female; -, negative control ear notch from a B6-Wt male; NT, no template; F, female; Het, heterozygote; Wt, wild type; M, male; Hemi, hemizygote; recomb., recombined loxP sites. Note: all blastocysts were also genotyped and found to be negative for 2loxP. L, 100 bp DNA ladder; NT, no template. (E) Blastocysts were harvested from B6129F1 mice and cultured to establish embryonic stem cells (ESCs). Genomic DNA amplification of ESCs resulted in detection of 1loxP heterozygous female cell lines, but no 1loxP hemizygous male cell lines. Statistical analysis showed that the female:male ratio was not significantly different from that expected but did show a trend towards less male cell lines. However, regardless of sex, or considering males only, the loxP:Wt ratios were significantly different from those expected, as only 15.8% of embryos carried a recombined 1loxP. (F) Blastocysts were harvested from B6129F1 mice and cultured to establish ESCs. Genomic DNA amplification of ESCs resulted in detection of 2loxP embryos of both sexes. Statistical analysis showed that the female:male ratio was not significantly different from that expected. Furthermore, regardless of sex, or considering males only, the loxP:Wt ratios were not significantly different from those expected, as 63% of cell lines carried a recombined 1loxP. Statistical analyses used the two-tailed binomial test for sex ratios and one-tailed binomial test for loxP:Wt as only loss of loxP embryos or cell lines was anticipated. Red, females; blue, males.

To discover when the 1loxP heterozygous embryos died and to eliminate the theoretical possibility that the 1loxP chromosome was not entering the germline of the first-generation 1loxP heterozygous females, we undertook timed pregnancy experiments. First, we harvested embryonic day (E) 9.5 embryos from B6-1loxP dams crossed to B6 sires (Fig. 3A), and our initial observation was that a portion of the decidua did not contain any embryonic tissue (embryo or yolk sac) (Fig. 3A,B). This ‘empty cup’ phenotype suggested that implantation occurred but the embryo failed to develop. Overall, we observed 15 empty decidua and 15 decidua from which we could recover E9.5 embryos within yolk sacs. The yolk sacs were genotyped for sex and *Taf1* exon 8 status, and the results were seven Wt female embryos and eight Wt male embryos. Neither 1loxP heterozygote females nor 1loxP hemizygote males were recovered at this stage of development.

Following this observation, E3.5 blastocysts were harvested, both from B6 mice as above and from hybrid B6129F1 mice using B6 dams crossed to 129 sires (Fig. 3C,D). Overall, we recovered a total of 55 blastocysts. Surprisingly, all four genotypes were recovered: 18 1loxP heterozygous female blastocysts, 15 Wt female blastocysts, nine 1loxP hemizygous male blastocysts and 13 Wt male blastocysts. A similar breakdown of genotypes was observed on both backgrounds, demonstrating that recombinant 1loxP mice are able to survive to the blastocyst stage and, as suggested by the E9.5 data above, are able to reach the implantation stage and induce uterine decidualisation.

We then went on to produce 1loxP and 2loxP ESCs from B6129F1 E3.5 blastocysts (Fig. 3E,F). From the experimental 1loxP cross, we recovered a total of 19 ESC lines: three 1loxP heterozygous female lines, 11 Wt female lines, zero 1loxP hemizygous male lines and five Wt male lines (Fig. 3E). From our observations, it appeared that 1loxP hemizygous male embryos in culture were not able to hatch and form viable cell lines. For the control 2loxP cross, we recovered a total of 14 ESC lines: five 2loxP heterozygous female lines, three Wt female lines, four 2loxP hemizygous male lines and two Wt male lines (Fig. 3F).

### Overall expression of *Taf1* mRNA brain transcripts is not different between genetic groups

To determine whether insertion of the loxP sites in 2loxP heterozygous, 2loxP homozygous or 1loxP heterozygous mice affected gene transcription, quantitative reverse-transcription PCR (RT-qPCR) was performed. The mouse *Taf1* gene encodes at least three known protein-coding transcripts including *Taf1*-201, *Taf1*-202 (canonical transcript) and *Taf1*-206 (Ensembl: ENSMUSG00000031314.19) (Fig. 1A). Cre-loxP recombination targets exon 8 and downstream microexon 8′, leading to induction of a stop codon in exon 9, for termination of further mRNA translation. Primers were designed against specific exons for detection of each of the *Taf1* transcripts: exon 7 (upstream of the deleted region), exon 8 (deleted region), exon 34, exon 34′ (targets the neuronal-specific *nTaf1* isoform; *Taf1*-206 variant) and exon 38 (targets *Taf1*-201 variant). In 1loxP heterozygous female mice, no transcripts from the deleted allele were detected in any PCR products spanning the deletion.

**Fig. 4.**
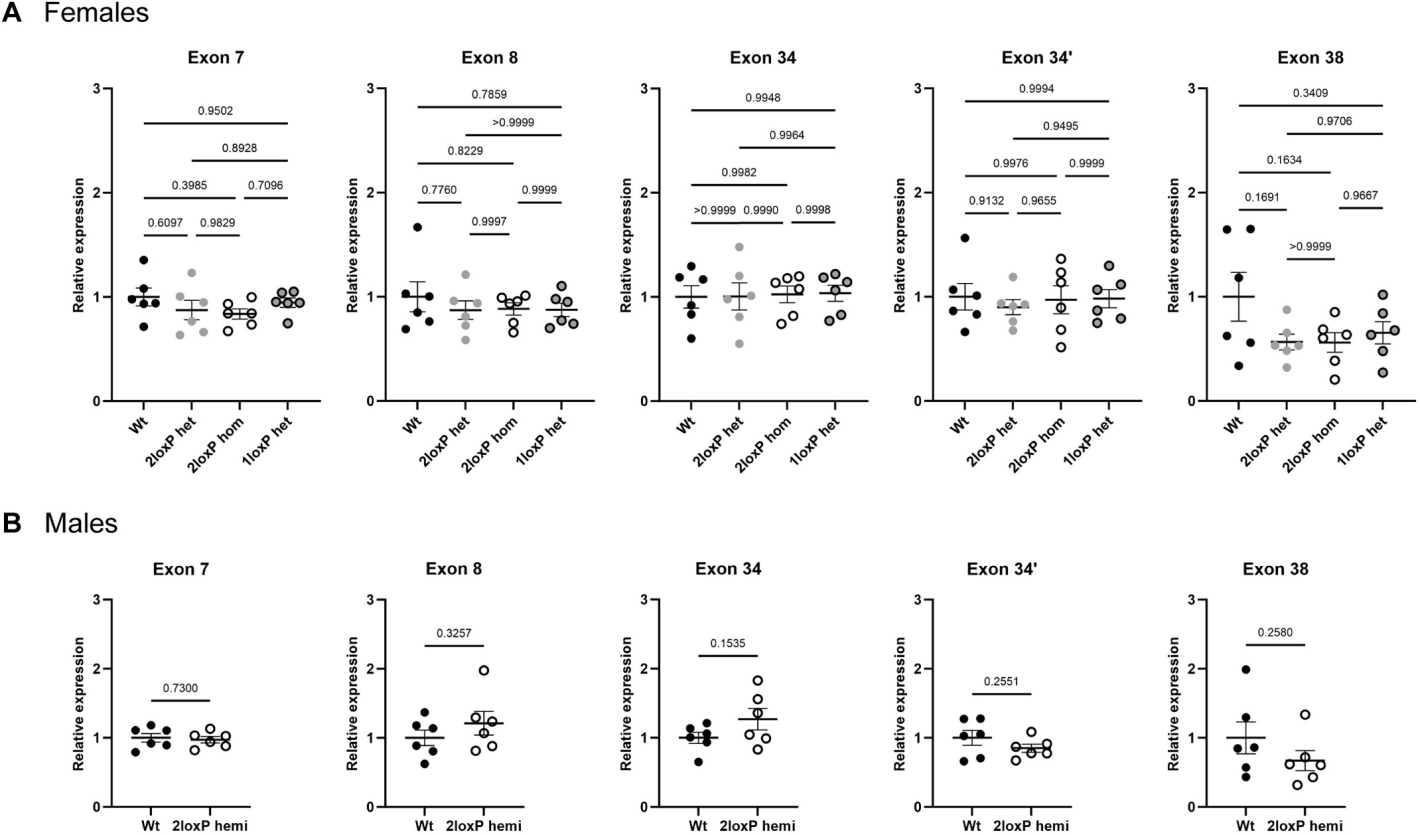
***Taf1* mRNA expression in the mouse brain.** RT-qPCR data are shown for (A) female and (B) male mice using the indicated primer pairs. The primer pairs targeted exons 7 (upstream of the deleted region), 8 (deleted region), 34, 34′ (targets the *nTaf1* isoform) and 38 (targets the *Taf1*-201 variant). For all PCRs, β-actin (*Actb*) was used as the internal control gene for Ct value normalisation. The relative expression was measured using the 2^−ΔΔCT^ method, then normalised to values for Wt mice. Mice were 6-7 months of age. Each dot represents one mouse (*n*=6/group). Error bars show mean±s.e.m. Statistical analysis using one-way ANOVA with Tukey's multiple comparison test was performed for A and unpaired two-tailed *t*-test for B; *P*-values are indicated. The experiment was replicated three times or more during the optimisation stage.

RT-qPCR analysis of female mouse brains revealed that there were no differences in the expression level of each of these transcripts detected at the respective exons with either 2loxP or 1loxP sites compared to that in Wt control mice (Fig. 4A). Similarly, 2loxP hemizygous male mice showed no differences in *Taf1* gene expression compared to that in Wt mice (Fig. 4B). Two to three RT-qPCR plates were run for each set of primers (*n*=2-3/genotype per plate) during the optimisation stage, resulting in reproducible cycle threshold (Ct) values with no differences between plates, which were normalised to results from Wt mice, then combined for analysis (total *n*=6/genotype). These findings suggest that there was no effect of insertion of the 2loxP sites into the mouse genome on the overall levels of *Taf1* mRNA expression and that 1loxP heterozygous female mice showed normal levels of *Taf1* mRNA expression.

### No variation in TAF1 protein expression in the brain in 1loxP heterozygous female mice

**Fig. 5.**
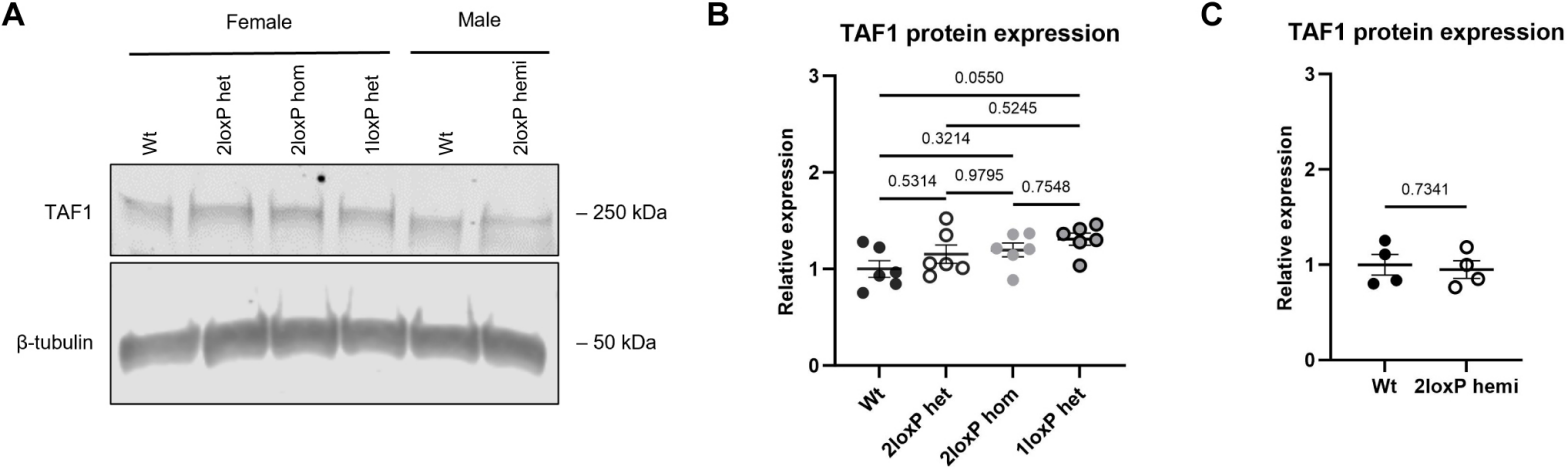
**TAF1 protein expression in the mouse brain.** (A-C) Comparison of TAF1 protein expression in non-recombinant mice, recombinant 1loxP heterozygous female mice and Wt littermates at 6-7 months of age. The presence of non-recombinant loxP sites did not alter TAF1 protein expression (A) and TAF1 abundance was not altered in 1loxP heterozygous female mice compared to that in littermates (2loxP heterozygous, 2loxP homozygous, 1loxP heterozygous and Wt mice; *n*=6/genotype) (B) or in 2loxP hemizygous male mice compared to that in Wt littermates (2loxP hemi, Wt; *n*=4/genotype) (C). Error bars show mean±s.e.m. Statistical analysis using one-way ANOVA with Tukey's multiple comparison test was performed for B and unpaired two-tailed *t*-test for C; *P*-values are indicated. The experiment was replicated three times or more during the optimisation stage.

The relative level of TAF1 protein expression was determined by western blot analysis of total brain lysates isolated from Wt, 2loxP heterozygous, 2loxP homozygous and 1loxP heterozygous female mice, and from Wt and 2loxP hemizygous male mice. No variation in TAF1 protein expression was observed among these genotypes, as detected with a TAF1-specific monoclonal antibody (Capponi et al., 2020) (Fig. 5; Table S5). Reproducible results were obtained through multiple blots and a representative blot is shown in Fig. 5A. The quantification shown in Fig. 5B is from two separate blots – expression levels were normalised to the Wt values, then combined – whereas samples were run for male mice on the same gel (Fig. 5C). This suggests that there is no impact of the loxP sites on TAF1 expression and that 1loxP heterozygous female mice express TAF1 at normal levels.

### *Taf1* deletion in heterozygous female mice does not affect nuclear TAF1 brain expression

**Fig. 6.**
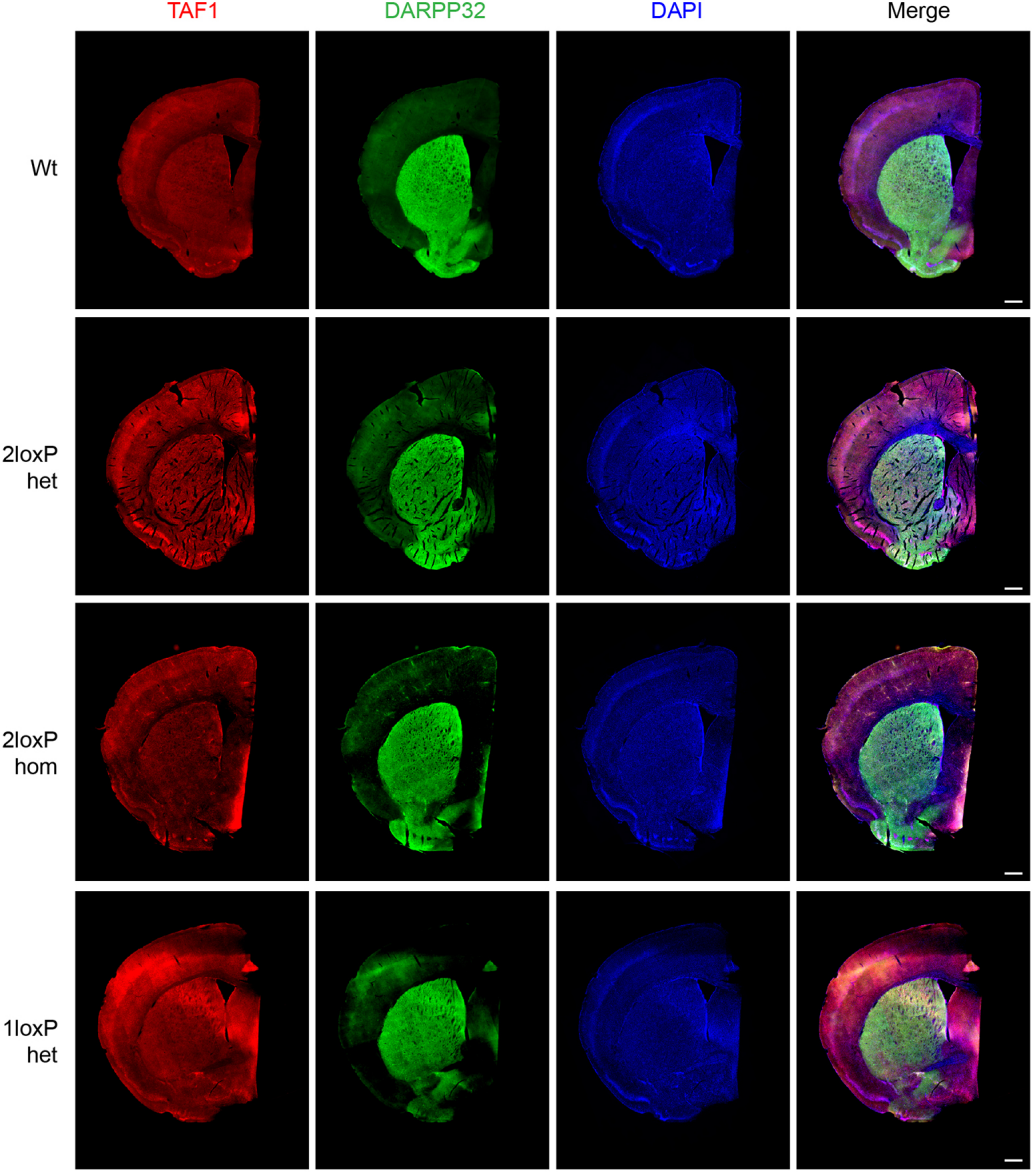
**TAF1 expression across the striatum and cortex.** Immunostaining is shown for 3- to 5-month-old female mice for the wild-type (Wt) condition and the genotypes 2loxP heterozygous (het), 2loxP homozygous (hom) and 1loxP het (following cre recombination, bred with EIIa-cre mice). TAF1 (red) was co-stained with DARPP32 (green), which indicates medium spiny neurons in the striatum, and DAPI (blue) was used to stain all nuclei. The experiment was replicated three times or more during the optimisation stage. Due to inadequate quality of some samples, *n*=1 image was obtained per genotype. Scale bars: 500 μm.

As western blotting revealed no significant differences in TAF1 expression across genetic groups, immunofluorescence staining was performed to visualise TAF1 expression in the female mouse brain. Initially, antibody optimisation and positive and negative controls were performed (Figs S1 and S2). Then, TAF1 expression was analysed across coronal sections showing the striatum and cortex (Fig. 6). An antibody against DARPP32 (encoded by *Ppp1r1b*) was used to mark the striatum, and TAF1 was found to be ubiquitously expressed in both the cortex and striatum as well as other regions including the corpus callosum. Multiple coronal sections were taken from each mouse and each brain section gave reproducible results. Overall, *Taf1* 1loxP heterozygous female mice showed similar ubiquitous expression of TAF1 as that in Wt mice. Of note, the total areas of the brain and striatal and cortical areas were quantified and showed no difference among genotypes (Fig. S3A-C).

**Fig. 7.**
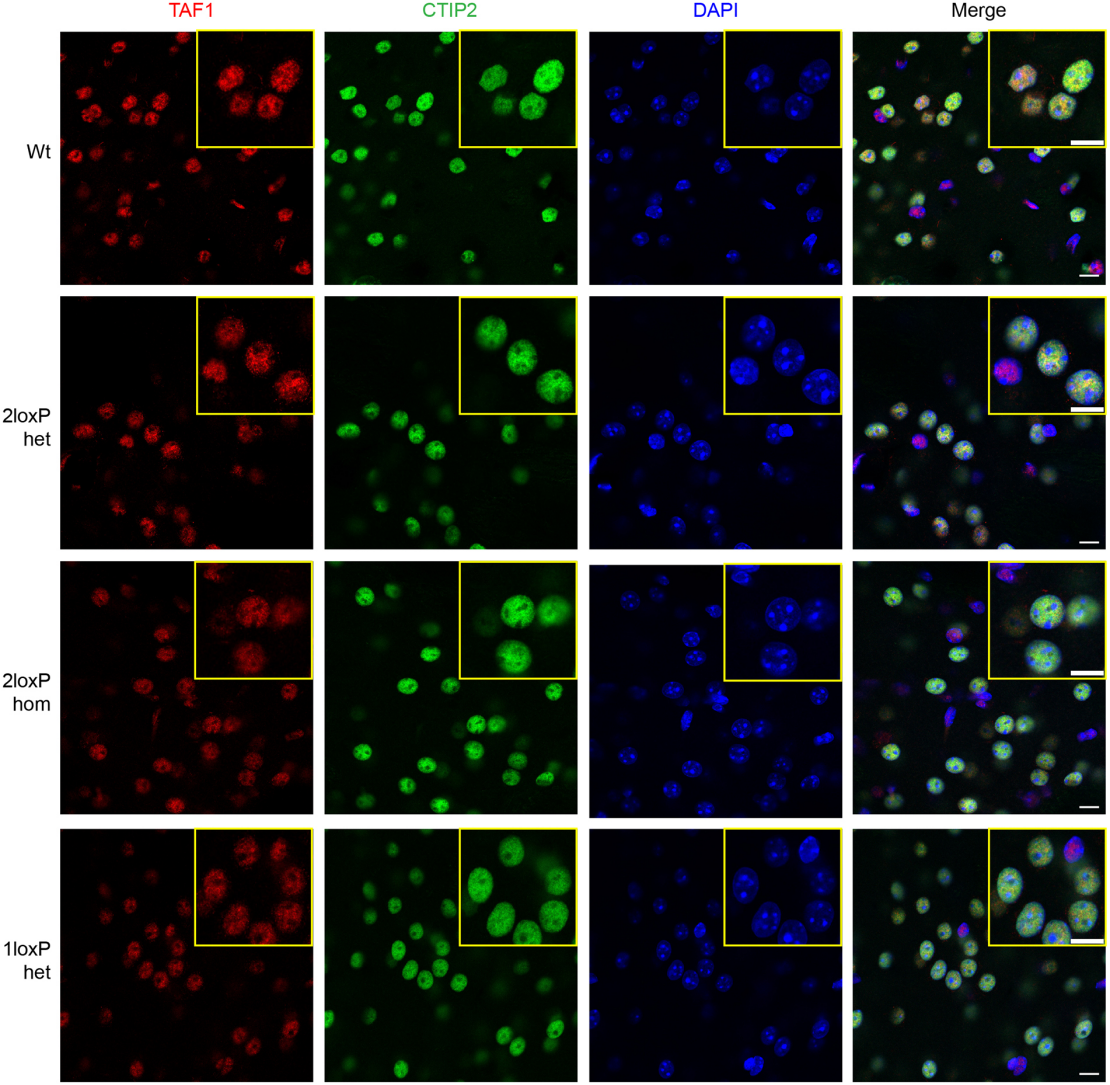
**TAF1 expression in nuclei in the striatum.** TAF1 expression (red) is shown for 3- to 5-month-old female mice for the wild type (Wt) condition and the genotypes 2loxP heterozygous (het), 2loxP homozygous (hom) and 1loxP het (following cre recombination, bred with EIIa-Cre mice). The anti-CTIP2 antibody (green) was used to co-stain medium spiny neuron nuclei and DAPI (blue) was used to stain all nuclei. Images are representative of *n*=3 mice per genetic group. The experiment was replicated three times or more during the optimisation stage. Quantification of nuclei stained for CTIP2 and TAF1 is shown in Fig. S4A,B. Scale bars: 10 μm.

TAF1 is a transcription factor with its main function and location in the nucleus. An antibody against CTIP2 (encoded by *Bcl11b*) was used to co-stain nuclei of medium spiny neurons in the striatum (Arlotta et al., 2008). All brains studied showed TAF1 staining that was specific to nuclei and all visible nuclei were stained for TAF1 (Fig. 7). TAF1 was highly expressed in medium spiny neurons as well as in other cells in the striatum in all groups (Fig. S4A). The 1loxP heterozygous female mice did not show differences in TAF1 staining when compared to the other genetic groups. This experiment was highly reproducible and similar results were obtained with three separate sets of mice from each genotype. All nuclei were stained for TAF1 in heterozygous mice (Fig. S4B), which indicated that TAF1 expression was active in each cell. Of note, the number of TAF1- and CTIP2-stained nuclei, expressed as a percentage of the total nuclei, was not different among genetic groups (Fig. S4A,B).

### Abnormal weight and movement in 1loxP heterozygous female mice

**Fig. 8.**
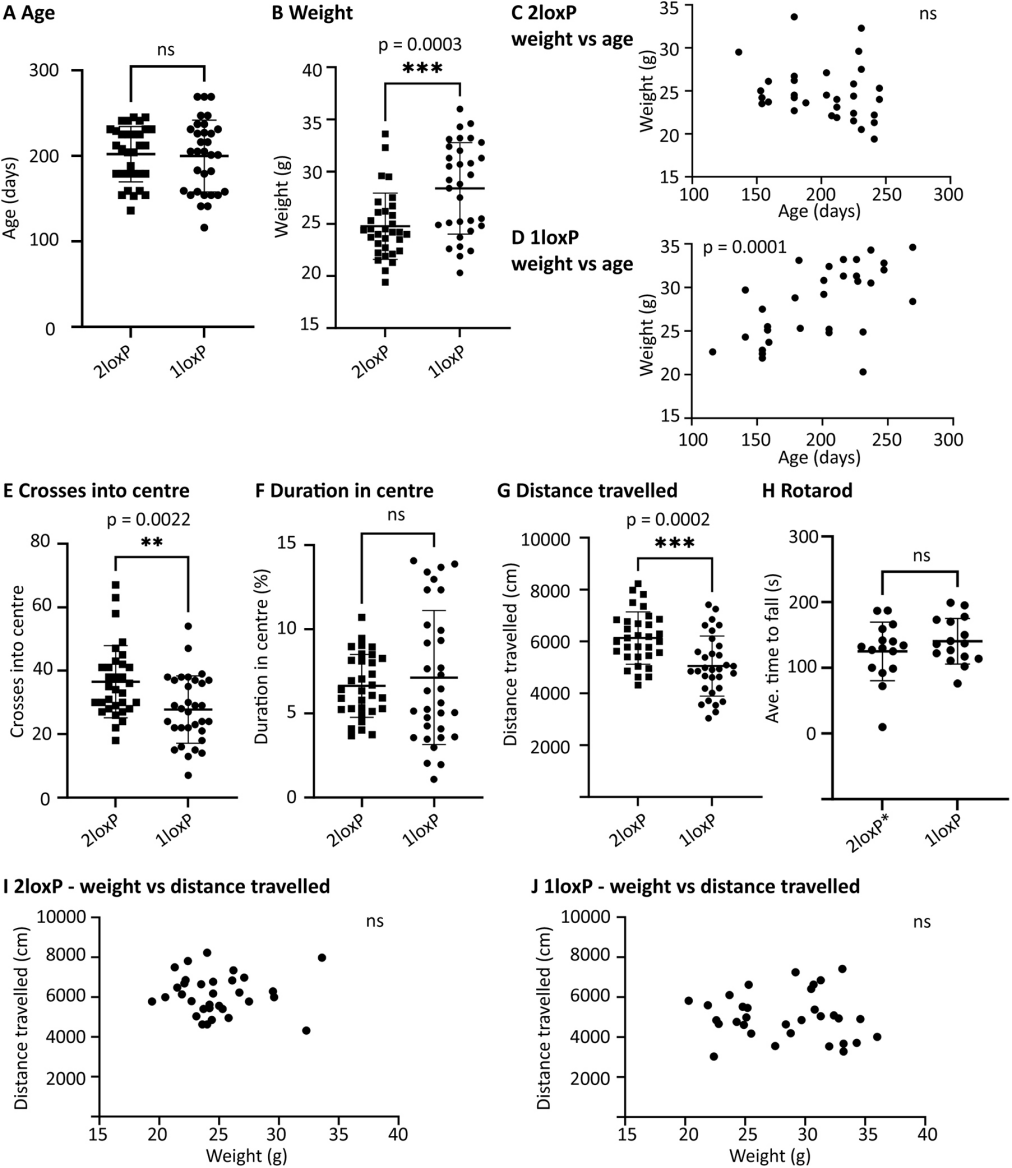
**Significant differences in weight and behaviour between 2loxP and 1loxP mice.** (A) Adult 2loxP and 1loxP female mice were age matched to 1loxP mice for behaviour testing (average age, 6.6 months) (*P*=0.8266). (B) However, when weighed, 1loxP mice were significantly heavier than 2loxP controls (*P*=0.0003). (C,D) The relationship between weight and age is shown. 2loxP control mice showed a non-significant negative correlation (*P*=0.1800, r=-0.2431) (C), whereas 1loxP experimental mice showed a significant positive correlation (*P*=0.0001, r=0.6267) (D). (E-G) Open-field testing. The number of crosses into the centre zone of the open field was significantly different, with 1loxP experimental mice crossing less (*P*=0.0022) (E). The duration in the centre zone was not significantly different between genotypes (*P*=0.5243) (F). The distance travelled in the open field was significantly different, with 1loxP experimental mice traveling less (*P*=0.0002) (G). (H) Rotarod testing. The mean latency to fall was not significantly different between genotypes (*P*=0.2835). (I,J) The relationship between distance travelled and weight is shown. No significant correlation was found: 2loxP control mice (*P*=0.9576, r=-0.009785) (I); 1loxP experimental mice (*P*=0.6456, r=-0.08451) (J). Each dot represents a mouse. 1loxP heterozygous females (1loxP): *n*=32 (A,B,D-G,I), *n*=16 (H). 2loxP heterozygous and homozygous females (2loxP): *n*=32 (A-C,E-G,I). 2loxP heterozygous females (2loxP*): *n*=16 (H). Error bars in A,B,E-H show mean±s.e.m. Statistical analysis was performed using unpaired two-tailed *t*-tests (A,B,E-H) or two-tailed correlations used to compute the Pearson's coefficient (r) (C,D,I,J). *P*-values are indicated. ns, not significant.

Behaviour analysis was performed with two separate groups at two separate times. Each group consisted of 32 female mice; the first group with 16 2loxP heterozygous controls and 16 1loxP heterozygous experimental mice was used for open-field and rotarod testing, and the second group with 16 2loxP homozygous controls and 16 1loxP heterozygous experimental mice was used for open-field testing only. One investigator performed all the experiments. For open-field testing, there was no significant impact of arena or trial. For rotarod testing, there was no significant impact of trial or lane. Furthermore, all genotypes were age matched, and the 2loxP heterozygous and 2loxP homozygous controls did not differ in any of the measurements (age, weight, crosses into centre, duration in centre or distance travelled). Therefore, data were pooled between the groups as presented in Fig. 8.

Mice from each genotype were age-matched adults (Fig. 8A); therefore, it was unexpected to observe that 1loxP experimental mice were significantly heavier than the 2loxP controls (Fig. 8B, pooled, *P*=0.0003; significant difference also observed in both separate groups).

At this age (range, 3-8 months; average, 6.6 months), which is beyond the period of development and prior to obesity associated with extreme age, we did not expect a significant change of weight with age, as observed in the 2loxP controls (Fig. 8C, pooled, *P*=0.1800, r=−0.2431; no significance observed in separate groups). Unexpectedly, we observed a significant increase in weight with age for the 1loxP experimental mice (Fig. 8D, pooled, *P*=0.0001, r=0.6267; significant difference also observed in both separate groups). Thus, both increased weight and increased weight with age were significant phenotypes of the 1loxP experimental mice.

In open-field testing, we observed that 1loxP mice significantly crossed into the centre zone less (Fig. 8E, pooled, *P*=0.0022; significant difference also observed in both separate groups) and travelled less (Fig. 8G, pooled, *P*=0.0002; significant difference in one group, trend in second group). These are two highly related data sets that both indicate less movement in the 1loxP mice. The duration in the centre zone was not significantly different between groups (Fig. 8F, pooled, *P*=0.5243; no significance observed in separate groups). In rotarod testing (tested in the first group only), no significant difference was observed in the average time to fall between the control and experimental mice (Fig. 8H, *P*=0.2835).

To examine the hypothesis that decreased movement in the 1loxP experimental mice was due to their increased weight, the relationship between the two measurements was examined. No correlation was found between distance travelled and weight in the 2loxP control mice (Fig. 8I, pooled, *P*=0.9576, r=−0.009785; no significance observed in separate groups) or in the 1loxP experimental mice (Fig. 8J, pooled, *P*=0.6456, r=−0.08451, no significance observed in separate groups). Thus, the hypothesis that increased weight made the 1loxP mice move less was not supported.

## DISCUSSION

Here, we generated and validated the first KO mutant mouse strain for the *Taf1* X chromosome gene, which encodes the largest subunit of the TFIID basal transcription factor causative of human disease (Gudmundsson et al., 2019; O'Rawe et al., 2015). Given the ubiquitous and critical role of this gene (Human Protein Atlas version 23.0, https://www.proteinatlas.org/ENSG00000147133-TAF1, accessed January 2024), lethality was a concern; thus, we made a cre-lox conditional allele (Gu et al., 1993). As this allele was created with CRISPR, an approach efficient in making the desired genomic change but well known to also result in unwanted genome events (Tan et al., 2015), we extensively sequenced (44.8 kb) the final allele and found it free of spurious mutations. To mimic the human disease situation and validate this new conditional allele, we bred the mice to a ubiquitous cre driver allele that is expressed early in development, resulting in germline recombination of the loxP sites.

We discovered that in this mouse model, *Taf1* deletion in males was embryonically lethal. Importantly, this result may provide the explanation as to why no null variants have been reported in humans. Interestingly, without *Taf1*, 1loxP male embryos did not die immediately, as might have been expected in the absence of such a critical transcription factor. Instead, embryos were able to develop to the blastocyst stage (E3.5, ∼140 cells) and induce uterine decidualisation. As this time frame is beyond the maternal effect (Li et al., 2010), it suggests that the role of TAF1 in mammals may be specialised to a subset of transcripts at this stage of embryonic development. These findings are in line with results from studies on KO of other TAFs in mice, such as *Taf7* (Gegonne et al., 2012), *Taf8* (Voss et al., 2000) and *Taf10* (Mohan et al., 2003), which also resulted in termination of development at early embryonic stages. Moreover, KO of *Taf8* or *Taf10* did not impede embryo hatching, decidualisation or trophectoderm viability, but prevented inner cell mass development (Mohan et al., 2003; Voss et al., 2000).

The blastocyst stage is when mouse ESCs can typically be derived. However, we were unable to establish 1loxP male ESCs, although we could establish ESCs from all other genotypes: Wt female and male, 2loxP heterozygous female and hemizygous male, and 1loxP heterozygous female. We anticipate that the 2loxP hemizygous male cell lines, when transfected with cre to induce loxP recombination and *Taf1* deletion, will be an important new mammalian tool for advancing our knowledge regarding the cellular role of TAF1.

Human heterozygous female carriers of *TAF1* variants demonstrate a spectrum of phenotypes from asymptomatic to dystonia–parkinsonism features (Giovenino et al., 2023; Gudmundsson et al., 2019; Lee et al., 2011; Vianna et al., 2020). Given that the 1loxP heterozygous female mice carried an even more severe mutation (null) than that observed in humans, and that in males, it was lethal, we anticipated that the females would be strongly affected. As this is a neurological disease, we studied the brains of *Taf1* 1loxP heterozygous females for gross structural abnormalities, overall expression by quantification of TAF1 mRNA and protein, and cellular localisation of the protein. To our surprise, the heterozygous female mouse brains had no differences in these parameters compared to Wt mouse brains. Building on the hypothesis based on observations in carrier human females that varying levels of skewed X inactivation may contribute to different disease severity (Evidente et al., 2004), we hypothesise that in the brains of these genetically identical mice, extreme skewing towards the non-mutant chromosome supported normal brain development and TAF1 expression and localisation. We speculate that in strongly symptomatic human carrier females, the skewing mechanism is not as robust as in mice, perhaps because it is negatively influenced by other genes in the genome, which vary among human individuals but not in the mice.

Exploring the phenotype of the 1loxP heterozygous female mice further, we discovered that they were indeed symptomatic. These mice exhibited a significant increase in weight, increase in weight with age, and reduced movement in the open-field test compared to control mice. Thus, despite hypothesizing that skewed X inactivation normalised the brains overall, we postulate that this mechanism was not totally effective even in mice. Thus, we suggest that a small subset of neurons in the brain were negatively impacted by the loss of *Taf1*, resulting in these phenotypes. Importantly, these mouse phenotypes suggest new avenues for clinical investigation of disease symptoms in human carrier females.

Beyond our studies, this new conditional cre-loxP mouse model for *Taf1* and the cell lines derived from it have the potential to reveal many aspects of TAF1 mammalian biology, which are still largely unknown. This includes, but is not limited to, the role of TAF1 dysregulation in cellular, developmental and human disease processes. Depending on the cre-expressing mouse strain this conditional allele is bred to, it can be used to create deletions in many different specific cell types or developmental times. Finally, and most critically, this mouse will be a platform for the advancement of therapeutics for TAF1 neurodevelopmental and neurodegenerative disease.

## MATERIALS AND METHODS

### Animal care and generation of cKO allele

All mice for all experiments were housed and bred in the pathogen-free Transgenic Animal facility at the Centre for Molecular Medicine and Therapeutics (CMMT) of the University of British Columbia (UBC). All mouse work was performed following protocols approved by the UBC Animal Care Committee (protocol numbers A19-0204 and A21-0140) in accordance with guidelines determined by the Canadian Council on Animal Care.

The online tool Benchling (https://benchling.com/crispr) was used to design guide RNAs using the *Streptococcus pyogenes* Cas9 nuclease to insert loxP sites surrounding exon 8, and also exon 8′ when present (Fig. 1). The best 5′ (intron 7) and 3′ (intron 8 or 8′) guides were selected based on the predicted on-target and off-target effects, and sense and anti-sense templates designed for the insertion of two loxP sites. Using the 10 µl Neon transfection system (Thermo Fisher Scientific, Waltham, MA, USA) to deliver CRISPR components into ESCs, as previously described in Mirjalili Mohanna et al. (2020), the top two 5′ and 3′ guides were tested in combination with four templates. Commercially synthesised CRISPR RNA (crRNA) and *trans*-activating crRNA (tracrRNA) (GenScript, Piscataway, NJ, USA) were annealed at a 1:1 ratio by incubating at 95°C for 5 min to generate guide RNA. The single-stranded oligodeoxynucleotide (ssODN) templates had 100 bp homology on each side of the Cas9 cleavage sites and the insertion of two loxP sites at the cut sites (Integrated DNA Technologies, Coralville, IA, USA). A purified, highly concentrated Cas9 nuclease protein with a nuclear localisation signal was used (CP02; PNA Bio, Thousand Oaks, CA, USA). A ribonucleoprotein complex was formed by combining Cas9 with annealed guide RNA, separately, by incubating at room temperature for 15 min. Immediately prior to electroporation, the ssODN template was added to the ribonucleoprotein complex and 3×10^5^ ESCs were used per reaction using the Neon optimisation setting 14. At 48 h post electroporation, cells were lysed in a tissue homogenisation buffer (THB) as previously described (Mirjalili Mohanna et al., 2020) and the best combination of 5′ guide, 3′ guide and template was determined by PCR using the 5′ loxP and 3′ loxP primers described in Table S2.

Optimal CRISPR guide Elizabeth M. Simpson 23 (cgEMS23) and 30 (cgEMS30) along with optimal template oligonucleotide EMS6395 (oEMS6395) (Table S1) were used to generate the *Taf1* 5′ loxP mouse via cytoplasmic injection, as previously described (Mirjalili Mohanna et al., 2020). In brief, 0.5 days post coitus, plugs were obtained by superovulation of C57BL/6J (B6) females [strain #000664, The Jackson Laboratory (JAX), Bar Harbor, ME, USA] mated with B6 males. Zygotes were harvested into EmbryoMax M2 medium (MR-015-D; MilliporeSigma, Burlington, MA, USA) and were treated with hyaluronidase (H272; MilliporeSigma) to remove cumulus cells. Zygotes were then cultured in potassium simplex optimisation medium (KSOM, MR-121; MilliporeSigma) until the time of injection. A CRISPR solution totalling 25 µl was prepared as above, with a final concentration of 25 ng/µl cgEMS23, 25 ng/µl cgEMS30, 120 ng/µl oEMS6395 and 50 ng/µl SpCas9 (CP01; PNA Bio) diluted in embryo-grade Tris-EDTA (TE) buffer (CytoSpring, Mountain View, CA, USA). The cytoplasm of one-cell mouse zygotes was injected using the XenoWorks digital microinjection system (Sutter Instruments, Novato, CA, USA). Post-microinjection zygotes were placed back into KSOM and incubated until transferred into day 0.5 post-coitus pseudopregnant CD-1 females (strain #022, Charles River Laboratories, Wilmington, ME, USA).

The resulting pups were characterised by ear notching at postnatal day 21. Ear notches were digested in THB with protein kinase and genotyped by PCR using the 5′ loxP and 3′ loxP primers described in Table S2. One male founder was recovered, which only contained the 5′ loxP site (Fig. 1C). This male passed the new allele on to offspring and was used to establish the *Taf1* 5′ loxP strain, which was then backcrossed to B6 for two to three generations (N2-N3) and used for a second round of CRISPR to introduce the 3′ loxP site.

Benchling was used as above to design three 3′ (intron 8 or 8′) guides and three templates, with 60 bp homology on each side of the Cas9 cleavage sites and the insertion of a loxP site. The three combinations were tested in ESCs and characterised using the 10 µl Neon transfection system as described above, and the optimal set was determined by PCR.

Optimal guide cgEMS23 and optimal template oEMS6414 (Table S1) were then used to generate *Taf1* 2loxP mice via i-GONAD electroporation following the Ford and Yamanaka protocol (Ford and Yamanaka, 2022; Gurumurthy et al., 2019). In brief, 0.5 days post coitus, plugs were obtained by crowding B6-*Taf1* 5′ loxP heterozygous or homozygous females for 10 days, followed by mating with B6-*Taf1* 5′ loxP hemizygous males. A CRISPR solution totalling 20 µl was prepared as above, with a final concentration of 30 µM cgEMS39, 40 µM oEMS6414, 1 µg/µl SpCas9 and 0.2% wt/vol Fast Green (for visualisation of injection success) diluted in Opti-MEM (Thermo Fisher Scientific). The oviduct was injected and immediately covered with a piece of sterile Kimwipe soaked in 1× PBS, and the Kimwipe-covered oviduct was placed between tweezer-like electrodes (LF650P3; BEX, Japan). Using the CUY21EDIT-II Electroporator (BEX), a square-wave pulse was applied with the following conditions: mode, square (mA); pattern, +/−; PdV, 80 V; PdA, 100 mA; Pd on, 5.00 ms; Pd off, 50 ms; PdN, 3; decay, 10%; decay type, log.

The resulting pups were characterised as above using the 3′ loxP primers described in Table S2. This resulted in three founders, one female and two males, which contained both the 3′ and 5′ loxP sites (Fig. 1D). The female and one male each passed the new allele to offspring, resulting in the new strains C57BL/6J-*Taf1^em1Ems^* (*Taf1^em1Ems^*) and C57BL/6J-*Taf1^em2Ems^* (*Taf1^em2Ems^*).

The numbers of mice used were determined by the need to obtain at least one founder capable of establishing the 2loxP strain. Statistical tests were not used. Mice were excluded only by genotype and the investigator was aware of the genotype.

The *Taf1* cKO mouse strain developed here, C57BL/6J-*Taf1^em2Ems^* (Mouse Genome Informatics MGI:6728059), is available from two non-profit distributors: the Mutant Mouse Resource and Research Centers (MMRRC strain 069592-JAX) and the European Mouse Mutant Archive (EMMA; EM:14890).

### Samplix indirect sequence capture

Samplix sequencing was performed as previously described (Devoy et al., 2021) using primers specific to this project (Table S3). High molecular mass genomic DNA from prospective founder mice was first encapsulated into picolitre-sized droplets using an Xdrop microfluidics device (Samplix). Fluorescent in-droplet PCR (dPCR) using the primers at the target locus labelled droplets harbouring target site DNA fragments, which were subsequently isolated via flow cytometry sorting and amplified via multiple displacement amplification, prior to Oxford nanopore sequencing. In cases of DNA degradation, enrichments obtained with both the main and backup primer sets were pooled and sequenced. The investigator was unaware of the mouse genotype during the analysis.

### Breeding of cKO allele to ubiquitous EIIa-cre

Standard mouse breeding strategies involved trios, two females and one male in a cage. Genotyping was by tail tip in newborn mice and by ear notch in older mice. To recombine and delete the *Taf1* gene, 2loxP mice were bred to B6.FVB-Tg(EIIa-cre)C5379Lmgd/J (Ella-cre, N10+N4 C57BL/6, JAX stock #003724). Genomic DNA amplification assays were developed to uniquely identify Wt, 2loxP and 1loxP alleles in all combinations in heterozygous, homozygous and hemizygous mice (Fig. 2C; Table S2).

The numbers of mice were determined by the needs of the subsequent experiments and are presented in Fig. 2. Statistical tests are also presented in Fig. 2 and the ‘Statistical analysis’ section below. No mice were excluded nor was the investigator unaware of the genotype.

### Timed matings, blastocyst genotyping and ESC generation

Timed pregnancies were achieved as previously described (de Leeuw et al., 2016), using crowded females, experienced studs and plug checking of females to determine the date of copulation. B6-*Taf1* 2loxP and B6-*Taf1* 1loxP heterozygous females were bred with either B6 or 129S1/SvImJ (JAX stock #002448) males, and on E9.5 or E3.5, pregnant dams were sacrificed by cervical dislocation and their uterus removed. At E9.5, decidua were isolated and dissected to remove the yolk sac/embryo. Decidua and yolk sacs/embryos were imaged using a Leica MZ125 microscope with a CoolSnap-Pro CF camera (Leica Microsystems, Wetzlar, Germany). Following imaging, yolk sacs were digested in THB and samples were genotyped for sex and loxP status using the primers in Table S2. At E3.5, single blastocysts were isolated and either used directly for genotyping or used for ESC generation.

DNA derived from a single E3.5 blastocyst was prepared and used for PCR according to the method described in Sakurai et al. (2014). Briefly, blastocysts were flushed from the uterine horns and cultured in EmbryoMax KSOM with 1/2 Amino Acids, glucose and Phenol Red (MilliporeSigma), individual blastocysts were transferred to a 0.2 ml thin-walled PCR tube, and 10 µl blastocyst lysis buffer [125 μg/ml proteinase K, 100 mM Tris-HCl (pH 8.3), 100 mM KCl, 0.02% gelatin, 0.45% Tween 20 and 60 μg/ml yeast tRNA (Ambion, Carlsbad, CA, USA)] was added. PCR tubes were then incubated at 56°C for 10 min, followed by incubation at 95°C for 10 min. Lysis was followed by whole-genome amplification using the REPLI-g Mini Kit (QIAGEN, Hilden, Germany) following the manufacturer's protocol. This was followed by standard genotyping for sex and loxP status using the primers in Table S2.

E3.5 blastocysts were used to derive ESC lines as previously described (Yang et al., 2009). Briefly, blastocysts were flushed from the uterine horns and cultured in EmbryoMax KSOM with 1/2 Amino Acids, glucose and Phenol Red for 3-5 h, and then transferred onto mitotically inactivated mouse embryonic feeders (MEFs) in 96-well plates containing KSR-ESC medium [Knockout DMEM (10829-018; Thermo Fisher Scientific) with 2 mM L-glutamine (25030-081; Thermo Fisher Scientific), 0.1 mM MEM nonessential amino acid solution (11140-050; Thermo Fisher Scientific), 1000 U/ml ESGRO LIF (ESG1107; MilliporeSigma) and 16% Knockout Serum Replacement (10828-028; Thermo Fisher Scientific)]. Blastocysts were cultured for 7-9 days in KSR-ESC medium, following which blastocysts that had hatched out of the zona pellucida and showed an inner cell mass were treated with 20 µl of 0.25% Trypsin-EDTA (25200-072; Thermo Fisher Scientific) for 5 min at 37°C. They were then triturated and inactivated with 30 µl of 0.5 mg/ml soybean trypsin inhibitor (17075-029; Thermo Fisher Scientific), and the solution was brought up to 200 µl with KSR-ESC medium and transferred individually to a 24-well MEF plate containing 1800 µl KSR-ESC medium (to a total volume of 2 ml). Beginning 4 days later, KSR-ESC medium was replaced with 25%, 50% and 75% FBS-ESC medium [DMEM (11960-069; Thermo Fisher Scientific) with 2 mM L-glutamine, 0.1 mM MEM nonessential amino acid solution, 1000 U/ml ESGRO LIF, 16% ES Cell Qualified fetal bovine serum (FBS, 16141-079; Thermo Fisher Scientific) and 0.01% β-mercaptoethanol (M-7522; MilliporeSigma)] to adapt the cells to FBS-containing medium. On day 7, the cells were trypsinised to one well of a 24-well plate containing 1 ml of 100% FBS-ESC medium, with daily medium replacement. Once confluent, wells containing ESC colonies were expanded for freezing (on MEFs) and for DNA extraction (on gelatin). Cells were washed and harvested in THB, and samples were genotyped for sex and loxP status using the primers in Table S2.

The numbers of female mice used in each timed pregnancy experiment were adjusted based on the expected embryo harvest and need to test loxP:Wt significance. A hypothesis of a 1:1 ratio allowed us to model mouse numbers for a significance of *P*=0.05. Embryo numbers and statistical tests are presented in Fig. 3 and in the ‘Statistical analysis’ section below. No embryos were excluded. The investigator was unaware of the genotype when dissecting and scoring E9.5 decidua as being empty or containing an embryo. They were also unaware of the genotype when doing PCR for E9.5 and E3.5 embryos and ESCs. Otherwise, the investigator was aware of the genotype.

### Brain RNA extraction and RT-qPCR

For each mouse, total RNA was extracted from one brain hemisphere using the RNeasy kit (QIAGEN) according to the manufacturer's instructions. Tissue was homogenised on ice using a TissueRuptor II (QIAGEN) in Buffer RLT with 1% β-mercaptoethanol. The final extracted RNA was eluted in DNase- and RNase-free water. The amounts of RNA were equalised and cDNA was generated using the SuperScript First-Strand Synthesis System for RT-PCR (11904-018; Thermo Fisher Scientific). RT-qPCR was undertaken to determine expression of all isoforms of *Taf1* (Table S4). Ct values were normalised by those of β-actin (*Actb*) as an endogenous control. Minus template controls were run for every sample for all reactions.

As this is the first *Taf1* KO mouse strain studied, there were no previous data on which to model effect size for significance of *P*=0.5. Thus, *n*=6 was calculated by the ‘resource equation’ method (Charan and Kantharia, 2013) and based on a previous publication showing a quantifiable effect of genetic KO by RT-qPCR with three to seven experimental mice (van den Hoogenhof et al., 2017). The experiment was repeated for individual mouse samples that were identified and excluded as outliers in the Grubb's outliers test, but no animals were excluded from the analysis. No randomisation was used and the investigator was aware of the genotype. The data met the assumptions for the ANOVA statistical test that was used.

### Protein isolation and western blotting

For protein isolation from whole-brain tissue, one brain hemisphere was dissected in ice-cold PBS before snap freezing. Samples were homogenised in ice-cold radioimmunoprecipitation assay buffer (150 mM sodium chloride, 50 mM Tris, 1% NP-40, 0.5% sodium deoxycholate, 0.1% sodium dodecyl sulphate) plus Protease Inhibitor Cocktail I (Millipore, Billerica, MA, USA) by mechanical disruption using a TissueRuptor II (QIAGEN). Homogenates were shaken on ice at 70 rpm for 2 h at 4°C, then centrifuged for 20 min at 15,000 ***g*** at 4°C. Supernatants were removed and stored at −80°C until use. Total protein concentration was determined by Bradford assay using Protein Assay Dye Reagent (Bio-Rad, Hercules, CA, USA). Equal amounts of total brain protein were denatured in LDS denaturing buffer (Thermo Fisher Scientific) and β-mercaptoethanol, and incubated at 95°C for 5 min prior to separation by SDS-PAGE gel electrophoresis.

Protein samples (40 μg) from individual mice were loaded onto precast NuPAGE 7% Tris-Acetate gels (Thermo Fisher Scientific) and electrophoresed at 80 V for 4 h in an ice bath using NuPAGE Tris-Acetate Running Buffer (Thermo Fisher Scientific). Proteins were transferred to 0.2 μm nitrocellulose membranes using a Trans-Blot Turbo transfer system (Bio-Rad) High Molecular Weight Program at 1.3 A for 30 min. Membranes were stained for total protein with 0.1% (w/v) Ponceau S in 1% (v/v) acetic acid for 5 min prior to blocking for 1 h at room temperature in Intercept PBS Blocking Buffer (LI-COR Biosciences, Lincoln, NE, USA). Membranes were incubated overnight in primary antibodies diluted in Intercept PBS Blocking Buffer (LI-COR Biosciences) at 4°C (anti-TAF1 177-4, 1:1000; anti-α-actinin, 1:2000; anti-β-tubulin, 1:10,000). All primary antibodies used are validated and specific monoclonal antibodies are described in Table S5. Membranes were then washed three times for 10 min in PBS containing 0.05% Tween-20 (PBS-T) and incubated for 1 h at room temperature with the relevant IRDye secondary antibody (LI-COR Biosciences) diluted in Intercept PBS Blocking Buffer. Finally, membranes were washed three times for 10 min in PBS-T and once for 10 min in PBS. Antibody binding was visualised using the Odyssey CLx Imaging System (LI-COR Biosciences) and ImageJ was used for band density analysis and total protein quantification.

The same mouse brains (other hemisphere) and *n* values were used as in the ‘Brain RNA extraction and RT-qPCR’ section above. Reproducible results were obtained through multiple blots and the experiment was repeated for mouse samples that showed large variation in protein expression, which, following quantification, were excluded as outliers in the Grubb's outliers test, but no animals were excluded from the analysis. No randomisation was used and the investigator was aware of the genotype.

Relative signal of the anti-TAF1 antibody compared to total protein loading and to two different internal loading controls (α-actinin and β-tubulin) was calculated and the values were normalised to the mean relative signal of control samples electrophoresed on the same gel. The mean of technical replicates was calculated and used for ANOVA, for which biological replicates were used as the experimental unit and the assumptions for this test were met.

### Immunofluorescence staining and quantification

For histological analyses, mice were perfused with PBS so that blood was completely removed. Mice were then perfused with 50 ml of 4% paraformaldehyde. Brains were removed and fixed in 4% paraformaldehyde for 24 h at 4°C. Samples were then washed twice in PBS and stored in PBS containing 0.05% sodium azide at 4°C. Brains were sectioned on a vibrating microtome (Leica VT1000S) at a thickness of 40 µm and stored in PBS containing 0.05% sodium azide at 4°C until staining.

For Fig. 6, free-floating sections were blocked and permeabilised in 5% goat serum with 0.25% Triton X-100 for 1 h. Sections were incubated in primary antibodies (anti-TAF1, 1:200; anti-DARPP32, 1:1000) in 5% goat serum overnight at 4°C. All antibodies used are described in Table S5. Negative control sections were incubated in 5% goat serum only (Fig. S2A). All samples, including the negative controls, were washed three times for 5 min in PBS and incubated in secondary antibody [goat anti-rabbit IgG (H+L) Alexa Fluor 564, 1:400; goat anti-mouse IgG (H+L) Alexa Fluor 488, 1:1000] for 1 h. For Fig. 7, a prior antigen-retrieval step was performed in which sections were boiled in Citric Acid-Based Antigen Unmasking Solution (H-3300-250; Vector Laboratories, Newark, CA, USA) for 20 min at 95°C, left to cool for 20 min, then washed in distilled water for 5 min. Sections were then blocked and permeabilised in 5% goat serum with 0.25% Triton X-100 and 1% BSA for 1 h. Sections were incubated in primary antibodies (anti-TAF1, 1:100; anti-CTIP2, 1:250) in 5% goat serum overnight at 4°C. Negative control sections were incubated in 5% goat serum only (Fig. S2B). Samples were washed three times for 5 min in PBS and incubated in secondary antibody [goat anti-rabbit IgG (H+L) Alexa Fluor 564, 1:400; goat anti-rat IgG (H+L) Alexa Fluor 488, 1:400] for 1 h. Samples were washed three times in PBS, then incubated in DAPI solution for 5 min. Samples were mounted with Prolong Gold Antifade (P10144, Vector Laboratories) and coverslipped. Slides were imaged at high magnification using a Zeiss LSM980 confocal microscope, and whole sections were imaged using an Axio Scan Z1 slide scanner.

To quantify the area of different brain regions, a Haematoxylin and Eosin staining kit (ab245880, Abcam) was used to stain serial coronal sections close to those in Fig. 6. ImageJ was used to quantify total brain area, including the striatum and cortex. To quantify the number of anti-CTIP2- and anti-TAF1-stained nuclei in Fig. 7, stained nuclei were counted as a percentage of the total nuclei labelled with DAPI. Quantification was performed using ImageJ.

We found little to no variability when two mice and three sections per genotype were studied. When differences were observed, defined by preestablished criteria such as photobleaching or overexposure, the image was excluded and the experiment repeated for that mouse with a Wt control for comparison. No randomisation was used and the investigator was aware of the genotype.

### Behaviour testing

The 2loxP heterozygous or 2loxP homozygous female control mice and 1loxP heterozygous female experimental mice were selected based on age matching to the 1loxP heterozygous females, and all mice were aged to adulthood. No mice within the age-matched groups were excluded. Two rounds of behaviour testing were carried out, each with 16 2loxP heterozygous or 2loxP homozygous female control mice and 16 1loxP heterozygous female experimental mice. The first round included both open-field and rotarod testing, and the second round only included open-field testing. Two weeks prior to the testing, mice were singly housed and constrained randomisation was used to assign them to a trial. Each trial consisted of four mice (*n*=2 2loxP and *n*=2 1loxP mice) and, within each trial, a random number generator was used to assign each mouse a number from 1 to 4, which indicated which of the four arenas (open field) or four lanes (rotarod) the mouse would be placed in. The day before each open-field trial, mice were weighed. For all experiments, the investigator was unaware of the genotype and identity of the mice during testing and to the genotypes of the groups during statistical analysis.

Open-field locomotion was evaluated under regular light conditions and quantified by video analysis from an overhead camera, using EthoVision XT 14.0 software (Noldus, Leesburg, VA, USA). Mice were allowed to acclimatise to the testing room for 10 min before initiating testing. The equipment was cleaned with ethanol before and between each trial. A single 10-min trial was conducted using B6 (black) mice in white 50 cm×50 cm×20 cm arenas, with four arenas per trial. The centre zone was defined as a square covering 16% of the total arena (20 cm×20 cm central square). The total distance travelled, duration of time in the centre and frequency of crossing into the centre were quantified by video analysis software.

Motor coordination was assessed by accelerating rotarod (UGO Basile, Italy) with training as previously described in Life et al. (2023). Briefly, training took place over three consecutive days, with three trials performed each day, separated by intervals of 1 h. In each trial, mice were placed on the rotarod at a fixed speed of 15 rpm for up to 2 min. Mice were then rested for 5 days before entering the testing phase. In the testing phase, mice were placed on the rotarod with acceleration set from 5 to 40 rpm over 5 min, with a readout of latency to fall. A total of three trials were performed in a single day, separated by intervals of 1 h. Latency to fall over the three trials was averaged and used for analysis.

As this is the first *Taf1* KO mouse strain studied, there were no previous data on which to model effect size for significance of *P*=0.5. Thus, mouse numbers were based on the authors' previous experience with mouse model behaviour testing (Abrahams et al., 2005; Hossain et al., 2004; Wong et al., 2010). The experiment was separated into two groups to test reproducibility over time (months) and we report reproducible results. The standard statistical tests used were the unpaired two-tailed *t*-test and the two-tailed correlation test, with further details given in the ‘Statistical analysis’ section.

### Statistical analysis

All tests were performed in GraphPad Prism 10, which was accessed November, 2023. For Figs 2 and 3, the binomial test was used to analyse the data as it is an exact test to compare two categories. For female:male ratios, an unpaired two-tailed test was used because the expected probability is symmetrical. However, for loxP:Wt ratios, an unpaired one-tailed test was used because the null hypothesis anticipated only the loss of loxP mice, embryos or cell lines. For Figs 4A and 5B, the ‘resource equation’ method was used to determine the number of experimental mice as it is appropriate when no previous data are available (Charan and Kantharia, 2013), such as for this new mouse model. For Figs 4B and 5C, the unpaired two-tailed *t*-test was used to compare male mouse groups. For these RT-qPCR and western blot quantifications, samples were excluded based on the Grubb's outliers test (alpha=0.05, two-tailed), which is the standard method used when there is one clear outlier in the dataset (Barnett et al., 1994; Iglewicz and Hoaglin, 1993). For Fig. 8A,B,E-H, the unpaired two-tailed *t*-test was used to quantify the difference between the means for each variable from the two groups. For Fig. 8C,D,I,J, the two-tailed correlation test was used to quantify the correlation between two variables. The value of the Pearson correlation coefficient (r) was calculated, in addition to a *P*-value indicative of a significant different from no correlation (r=0).

## Supplementary Material

10.1242/dmm.050741_sup1 Supplementary information

## References

[DMM050741C1] Abrahams, B. S., Kwok, M. C. H., Trinh, E., Budaghzadeh, S., Hossain, S. M. and Simpson, E. M. (2005). Pathological aggression in “fierce” mice corrected by human nuclear receptor 2E1. *J. Neurosci.* 25, 6263-6270. 10.1523/JNEUROSCI.4757-04.2005 16000615 PMC6725287

[DMM050741C2] Aneichyk, T., Hendriks, W. T., Yadav, R., Shin, D., Gao, D., Vaine, C. A., Collins, R. L., Domingo, A., Currall, B., Stortchevoi, A. et al. (2018). Dissecting the causal mechanism of X-Linked Dystonia-Parkinsonism by integrating genome and transcriptome assembly. *Cell* 172, 897-909.e21. 10.1016/j.cell.2018.02.011 29474918 PMC5831509

[DMM050741C3] Arlotta, P., Molyneaux, B. J., Jabaudon, D., Yoshida, Y. and Macklis, J. D. (2008). Ctip2 controls the differentiation of medium spiny neurons and the establishment of the cellular architecture of the striatum. *J. Neurosci.* 28, 622-632. 10.1523/JNEUROSCI.2986-07.2008 18199763 PMC6670353

[DMM050741C4] Barnett, V., Lewis, T. and Rothamsted, V. (1994). *Outliers in Statistical Data*. John Wiley & Sons.

[DMM050741C5] Bhuiyan, T. and Timmers, H. T. M. (2019). Promoter recognition: putting TFIID on the spot. *Trends Cell Biol.* 29, 752-763. 10.1016/j.tcb.2019.06.004 31300188

[DMM050741C6] Blondal, T., Gamba, C., Møller Jagd, L., Su, L., Demirov, D., Guo, S., Johnston, C. M., Riising, E. M., Wu, X., Mikkelsen, M. J. et al. (2021). Verification of CRISPR editing and finding transgenic inserts by Xdrop indirect sequence capture followed by short- and long-read sequencing. *Methods* 191, 68-77. 10.1016/j.ymeth.2021.02.003 33582298

[DMM050741C7] Bragg, D. C., Mangkalaphiban, K., Vaine, C. A., Kulkarni, N. J., Shin, D., Yadav, R., Dhakal, J., Ton, M.-L., Cheng, A., Russo, C. T. et al. (2017). Disease onset in X-linked dystonia-parkinsonism correlates with expansion of a hexameric repeat within an SVA retrotransposon in TAF1. *Proc. Natl. Acad. Sci. USA* 114, E11020-E11028. 10.1073/pnas.1712526114 29229810 PMC5754783

[DMM050741C8] Bragg, D. C., Sharma, N. and Ozelius, L. J. (2019). X-linked Dystonia-Parkinsonism: recent advances. *Curr. Opin. Neurol.* 32, 604-609. 10.1097/WCO.0000000000000708 31116117 PMC7243267

[DMM050741C9] Capponi, S., Stöffler, N., Irimia, M., van Schaik, F. M. A., Ondik, M. M., Biniossek, M. L., Lehmann, L., Mitschke, J., Vermunt, M. W., Creyghton, M. P. et al. (2020). Neuronal-specific microexon splicing of TAF1 mRNA is directly regulated by SRRM4/nSR100. *RNA Biol.* 17, 62-74. 10.1080/15476286.2019.1667214 31559909 PMC6948980

[DMM050741C10] Charan, J. and Kantharia, N. D. (2013). How to calculate sample size in animal studies? *J. Pharmacol. Pharmacother.* 4, 303-306. 10.4103/0976-500X.119726 24250214 PMC3826013

[DMM050741C11] Cheng, H., Capponi, S., Wakeling, E., Marchi, E., Li, Q., Zhao, M., Weng, C., Piatek, S. G., Ahlfors, H., Kleyner, R. et al. (2019). Missense variants in TAF1 and developmental phenotypes: challenges of determining pathogenicity. *Hum. Mutat.* 41, 1075. 10.1002/humu.23936 PMC7187541 31646703

[DMM050741C12] Cirnaru, M. D., Creus-Muncunill, J., Nelson, S., Lewis, T. B., Watson, J., Ellerby, L. M., Gonzalez-Alegre, P. and Ehrlich, M. E. (2021). Striatal cholinergic dysregulation after neonatal decrease in X-linked dystonia Parkinsonism-related TAF1 isoforms. *Mov. Disord.* 36, 2780-2794. 10.1002/mds.28750 34403156

[DMM050741C13] de Leeuw, C. N., Korecki, A. J., Berry, G. E., Hickmott, J. W., Lam, S. L., Lengyell, T. C., Bonaguro, R. J., Borretta, L. J., Chopra, V., Chou, A. Y. et al. (2016). rAAV-compatible MiniPromoters for restricted expression in the brain and eye. *Mol. Brain* 9, 52. 10.1186/s13041-016-0232-4 27164903 PMC4862195

[DMM050741C14] Deng, X., Berletch, J. B., Nguyen, D. K. and Disteche, C. M. (2014). X chromosome regulation: diverse patterns in development, tissues and disease. *Nat. Rev. Genet.* 15, 367-378. 10.1038/nrg3687 24733023 PMC4117651

[DMM050741C15] Devoy, A., Price, G., De Giorgio, F., Bunton-Stasyshyn, R., Thompson, D., Gasco, S., Allan, A., Codner, G. F., Nair, R. R., Tibbit, C. et al. (2021). Generation and analysis of innovative genomically humanized knockin SOD1, TARDBP (TDP-43), and FUS mouse models. *iScience* 24, 103463. 10.1016/j.isci.2021.103463 34988393 PMC8710557

[DMM050741C16] Domingo, A., Lee, L. V., Brüggemann, N., Freimann, K., Kaiser, F. J., Jamora, R. D. G., Rosales, R. L., Klein, C. and Westenberger, A. (2014). Woman with x-linked recessive dystonia-parkinsonism: clue to the epidemiology of parkinsonism in Filipino women? *JAMA Neurol.* 71, 1177-1180. 10.1001/jamaneurol.2014.56 25004170

[DMM050741C17] Eppig, J. T., Smith, C. L., Blake, J. A., Ringwald, M., Kadin, J. A., Richardson, J. E. and Bult, C. J. (2017). Mouse Genome Informatics (MGI): resources for mining mouse genetic, genomic, and biological data in support of primary and translational research. *Methods Mol. Biol.* 1488, 47-73. 10.1007/978-1-4939-6427-7_3 27933520

[DMM050741C18] Evidente, V. G. H., Gwinn-Hardy, K., Hardy, J., Hernandez, D. and Singleton, A. (2002). X-linked dystonia (“Lubag”) presenting predominantly with parkinsonism: a more benign phenotype? *Mov. Disord.* 17, 200-202. 10.1002/mds.1263 11835466

[DMM050741C19] Evidente, V. G. H., Nolte, D., Niemann, S., Advincula, J., Mayo, M. C., Natividad, F. F. and Müller, U. (2004). Phenotypic and molecular analyses of X-linked dystonia-parkinsonism (“Lubag”) in women. *Arch. Neurol.* 61, 1956-1959. 10.1001/archneur.61.12.1956 15596620

[DMM050741C20] Ford, M. J. and Yamanaka, Y. (2022). Reprogramming mouse oviduct epithelial cells using in vivo electroporation and CRISPR/Cas9-mediated genetic manipulation. *Methods Mol. Biol.* 2429, 367-377. 10.1007/978-1-0716-1979-7_24 35507174

[DMM050741C21] Gegonne, A., Tai, X., Zhang, J., Wu, G., Zhu, J., Yoshimoto, A., Hanson, J., Cultraro, C., Chen, Q.-R., Guinter, T. et al. (2012). The general transcription factor TAF7 is essential for embryonic development but not essential for the survival or differentiation of mature T cells. *Mol. Cell. Biol.* 32, 1984-1997. 10.1128/MCB.06305-11 22411629 PMC3347399

[DMM050741C22] Giovenino, C., Trajkova, S., Pavinato, L., Cardaropoli, S., Pullano, V., Ferrero, E., Sukarova-Angelovska, E., Carestiato, S., Salmin, P., Rinninella, A. et al. (2023). Skewed X-chromosome inactivation in unsolved neurodevelopmental disease cases can guide re-evaluation For X-linked genes. *Eur. J. Hum. Genet.* 31, 1228-1236. 10.1038/s41431-023-01324-w 36879111 PMC10620389

[DMM050741C23] Goto, S., Lee, L. V., Munoz, E. L., Tooyama, I., Tamiya, G., Makino, S., Ando, S., Dantes, M. B., Yamada, K., Matsumoto, S. et al. (2005). Functional anatomy of the basal ganglia in X-linked recessive dystonia-parkinsonism. *Ann. Neurol.* 58, 7-17. 10.1002/ana.20513 15912496

[DMM050741C24] Gu, H., Zou, Y.-R. and Rajewsky, K. (1993). Independent control of immunoglobulin switch recombination at individual switch regions evidenced through Cre-loxP-mediated gene targeting. *Cell* 73, 1155-1164. 10.1016/0092-8674(93)90644-6 8513499

[DMM050741C25] Gudmundsson, S., Wilbe, M., Filipek-Górniok, B., Molin, A.-M., Ekvall, S., Johansson, J., Allalou, A., Gylje, H., Kalscheuer, V. M., Ledin, J. et al. (2019). TAF1, associated with intellectual disability in humans, is essential for embryogenesis and regulates neurodevelopmental processes in zebrafish. *Sci. Rep.* 9, 10730. 10.1038/s41598-019-46632-8 31341187 PMC6656882

[DMM050741C26] Gurumurthy, C. B., Sato, M., Nakamura, A., Inui, M., Kawano, N., Islam, M. A., Ogiwara, S., Takabayashi, S., Matsuyama, M., Nakagawa, S. et al. (2019). Creation of CRISPR-based germline-genome-engineered mice without ex vivo handling of zygotes by i-GONAD. *Nat. Protoc.* 14, 2452-2482. 10.1038/s41596-019-0187-x 31341289

[DMM050741C27] Hossain, S. M., Wong, B. K. Y. and Simpson, E. M. (2004). The dark phase improves genetic discrimination for some high throughput mouse behavioral phenotyping. *Genes Brain Behav.* 3, 167-177. 10.1111/j.1601-183x.2004.00069.x 15140012

[DMM050741C28] Hurst, S. E., Liktor-Busa, E., Moutal, A., Parker, S., Rice, S., Szelinger, S., Senner, G., Hammer, M. F., Johnstone, L., Ramsey, K. et al. (2018). A novel variant in TAF1 affects gene expression and is associated with X-linked TAF1 intellectual disability syndrome. *Neuron Signal.* 2, NS20180141. 10.1042/NS20180141 PMC7373232 32714589

[DMM050741C29] Huynh, K. D. and Lee, J. T. (2005). X-chromosome inactivation: a hypothesis linking ontogeny and phylogeny. *Nat. Rev. Genet.* 6, 410-418. 10.1038/nrg1604 15818384

[DMM050741C30] Iglewicz, B. and Hoaglin, D. C. (1993). *How to Detect and Handle Outliers*. American Society for Quality Control.

[DMM050741C31] Irvin, J. D. and Pugh, B. F. (2006). Genome-wide transcriptional dependence on TAF1 functional domains. *J. Biol. Chem.* 281, 6404-6412. 10.1074/jbc.M513776200 16407318

[DMM050741C32] Ito, N., Hendriks, W. T., Dhakal, J., Vaine, C. A., Liu, C., Shin, D., Shin, K., Wakabayashi-Ito, N., Dy, M., Multhaupt-Buell, T. et al. (2016). Decreased N-TAF1 expression in X-linked dystonia-parkinsonism patient-specific neural stem cells. *Dis. Model. Mech.* 9, 451-462. 10.1242/dmm.022590 26769797 PMC4852502

[DMM050741C33] Janakiraman, U., Yu, J., Moutal, A., Chinnasamy, D., Boinon, L., Batchelor, S. N., Anandhan, A., Khanna, R. and Nelson, M. A. (2019). TAF1-gene editing alters the morphology and function of the cerebellum and cerebral cortex. *Neurobiol. Dis.* 132, 104539. 10.1016/j.nbd.2019.104539 31344492 PMC7197880

[DMM050741C34] Kaloff, C., Anastassiadis, K., Ayadi, A., Baldock, R., Beig, J., Birling, M.-C., Bradley, A., Brown, S. D. M., Bürger, A., Bushell, W. et al. (2017). Genome wide conditional mouse knockout resources. *Drug Discov. Today: Dis. Model.* 20, 3-12. 10.1016/j.ddmod.2017.08.002 PMC11315453 39132094

[DMM050741C35] Lakso, M., Pichel, J. G., Gorman, J. R., Sauer, B., Okamoto, Y., Lee, E., Alt, F. W. and Westphal, H. (1996). Efficient in vivo manipulation of mouse genomic sequences at the zygote stage. *Proc. Natl. Acad. Sci. USA* 93, 5860-5865. 10.1073/pnas.93.12.5860 8650183 PMC39152

[DMM050741C36] Lee, L. V., Munoz, E. L., Tan, K. T. and Reyes, M. T. (2001). Sex linked recessive dystonia parkinsonism of Panay, Philippines (XDP). *Mol. Pathol.* 54, 362-368. 10.1136/mp.54.6.362 11724910 PMC1187125

[DMM050741C37] Lee, L. V., Rivera, C., Teleg, R. A., Dantes, M. B., Pasco, P. M. D., Jamora, R. D. G., Arancillo, J., Villareal-Jordan, R. F., Rosales, R. L., Demaisip, C. et al. (2011). The unique phenomenology of sex-linked dystonia parkinsonism (XDP, DYT3, “Lubag”). *Int. J. Neurosci.* 121, 3-11. 10.3109/00207454.2010.526728 21047175

[DMM050741C38] Li, L., Zheng, P. and Dean, J. (2010). Maternal control of early mouse development. *Development* 137, 859-870. 10.1242/dev.039487 20179092 PMC2834456

[DMM050741C39] Life, B., Petkau, T. L., Cruz, G. N. F., Navarro-Delgado, E. I., Shen, N., Korthauer, K. and Leavitt, B. R. (2023). FTD-associated behavioural and transcriptomic abnormalities in ‘humanized’ progranulin-deficient mice: a novel model for progranulin-associated FTD. *Neurobiol. Dis.* 182, 106138. 10.1016/j.nbd.2023.106138 37105261

[DMM050741C40] Luo, L., Ambrozkiewicz, M. C., Benseler, F., Chen, C., Dumontier, E., Falkner, S., Furlanis, E., Gomez, A. M., Hoshina, N., Huang, W.-H. et al. (2020). Optimizing nervous system-specific gene targeting with Cre driver lines: prevalence of germline recombination and influencing factors. *Neuron* 106, 37-65.e5. 10.1016/j.neuron.2020.01.008 32027825 PMC7377387

[DMM050741C41] Makino, S., Kaji, R., Ando, S., Tomizawa, M., Yasuno, K., Goto, S., Matsumoto, S., Tabuena, M. D., Maranon, E., Dantes, M. et al. (2007). Reduced neuron-specific expression of the TAF1 gene is associated with X-linked dystonia-parkinsonism. *Am. J. Hum. Genet.* 80, 393-406. 10.1086/512129 17273961 PMC1821114

[DMM050741C42] Malik, S. and Roeder, R. G. (2023). Regulation of the RNA polymerase II pre-initiation complex by its associated coactivators. *Nat. Rev. Genet.* 24, 767-782. 10.1038/s41576-023-00630-9 37532915 PMC11088444

[DMM050741C43] Mirjalili Mohanna, S. Z., Hickmott, J. W., Lam, S. L., Chiu, N. Y., Lengyell, T. C., Tam, B. M., Moritz, O. L. and Simpson, E. M. (2020). Germline CRISPR/Cas9-mediated gene editing prevents vision loss in a novel mouse model of aniridia. *Mol. Ther. Methods Clin. Dev.* 17, 478-490. 10.1016/j.omtm.2020.03.002 32258211 PMC7114625

[DMM050741C44] Mohan, W. S., Jr, Scheer, E., Wendling, O., Metzger, D. and Tora, L. (2003). TAF10 (TAF(II)30) is necessary for TFIID stability and early embryogenesis in mice. *Mol. Cell. Biol.* 23, 4307-4318. 10.1128/MCB.23.12.4307-4318.2003 12773572 PMC156135

[DMM050741C45] O'Rawe, J. A., Wu, Y., Dörfel, M. J., Rope, A. F., Au, P. Y. B., Parboosingh, J. S., Moon, S., Kousi, M., Kosma, K., Smith, C. S. et al. (2015). TAF1 variants are associated with dysmorphic features, intellectual disability, and neurological manifestations. *Am. J. Hum. Genet.* 97, 922-932. 10.1016/j.ajhg.2015.11.005 26637982 PMC4678794

[DMM050741C46] Rakovic, A., Domingo, A., Grütz, K., Kulikovskaja, L., Capetian, P., Cowley, S. A., Lenz, I., Brüggemann, N., Rosales, R., Jamora, D. et al. (2018). Genome editing in induced pluripotent stem cells rescues TAF1 levels in X-linked dystonia-parkinsonism. *Mov. Disord.* 33, 1108-1118. 10.1002/mds.27441 30153385

[DMM050741C47] Rosales, R. L. (2010). X-linked dystonia parkinsonism: clinical phenotype, genetics and therapeutics. *J. Mov. Disord.* 3, 32-38. 10.14802/jmd.10009 24868378 PMC4027667

[DMM050741C48] Sakurai, T., Watanabe, S., Kamiyoshi, A., Sato, M. and Shindo, T. (2014). A single blastocyst assay optimized for detecting CRISPR/Cas9 system-induced indel mutations in mice. *BMC Biotechnol.* 14, 69. 10.1186/1472-6750-14-69 25042988 PMC4118159

[DMM050741C49] Tan, E. P., Li, Y., Del Castillo Velasco-Herrera, M., Yusa, K. and Bradley, A. (2015). Off-target assessment of CRISPR-Cas9 guiding RNAs in human iPS and mouse ES cells. *Genesis* 53, 225-236. 10.1002/dvg.22835 25378133

[DMM050741C50] van den Hoogenhof, M. M. G., van der Made, I., Beqqali, A., de Groot, N. E., Damanafshan, A., van Oort, R. J., Pinto, Y. M. and Creemers, E. E. (2017). The RNA-binding protein Rbm38 is dispensable during pressure overload-induced cardiac remodeling in mice. *PLoS ONE* 12, e0184093. 10.1371/journal.pone.0184093 28850611 PMC5574583

[DMM050741C51] Vianna, E. Q., Piergiorge, R. M., Gonçalves, A. P., dos Santos, J. M., Calassara, V., Rosenberg, C., Krepischi, A. C. V., Boy da Silva, R. T., dos Santos, S. R., Ribeiro, M. G. et al. (2020). Understanding the landscape of X-linked variants causing intellectual disability in females through extreme X chromosome inactivation skewing. *Mol. Neurobiol.* 57, 3671-3684. 10.1007/s12035-020-01981-8 32564284

[DMM050741C52] Voss, A. K., Thomas, T., Petrou, P., Anastassiadis, K., Schöler, H. and Gruss, P. (2000). Taube nuss is a novel gene essential for the survival of pluripotent cells of early mouse embryos. *Development* 127, 5449-5461. 10.1242/dev.127.24.5449 11076765

[DMM050741C53] Walker, A. K., Shi, Y. and Blackwell, T. K. (2004). An extensive requirement for transcription factor IID-specific TAF-1 in Caenorhabditis elegans embryonic transcription. *J. Biol. Chem.* 279, 15339-15347. 10.1074/jbc.M310731200 14726532

[DMM050741C54] Warfield, L., Ramachandran, S., Baptista, T., Devys, D., Tora, L. and Hahn, S. (2017). Transcription of nearly all yeast RNA polymerase II-transcribed genes is dependent on transcription factor TFIID. *Mol. Cell* 68, 118-129.e5. 10.1016/j.molcel.2017.08.014 28918900 PMC5679267

[DMM050741C55] Wefers, B., Wurst, W. and Kühn, R. (2011). Design and generation of gene-targeting vectors. *Curr. Protoc. Mouse Biol.* 1, 199-211. 10.1002/9780470942390.mo100179 26068993

[DMM050741C56] Wong, B. K. Y., Hossain, S. M., Trinh, E., Ottmann, G. A., Budaghzadeh, S., Zheng, Q. Y. and Simpson, E. M. (2010). Hyperactivity, startle reactivity and cell-proliferation deficits are resistant to chronic lithium treatment in adult Nr2e1(frc/frc) mice. *Genes Brain Behav.* 9, 681-694. 10.1111/j.1601-183X.2010.00602.x 20497236 PMC3292041

[DMM050741C57] Yang, G. S., Banks, K. G., Bonaguro, R. J., Wilson, G., Dreolini, L., de Leeuw, C. N., Liu, L., Swanson, D. J., Goldowitz, D., Holt, R. A. et al. (2009). Next generation tools for high-throughput promoter and expression analysis employing single-copy knock-ins at the Hprt1 locus. *Genomics* 93, 196-204. 10.1016/j.ygeno.2008.09.014 18950699

