# Supplementary material for: Taf1 knockout is lethal in embryonic male mice and heterozygous females show weight and movement disorders
Source: Dis Model Mech. 2024 Jul 10;17(7):dmm050741. doi: 10.1242/dmm.050741 (PMC11261634; doi:10.1242/dmm.050741)
Supplement: Supplementary information [file dmm-17-050741-s1.pdf]

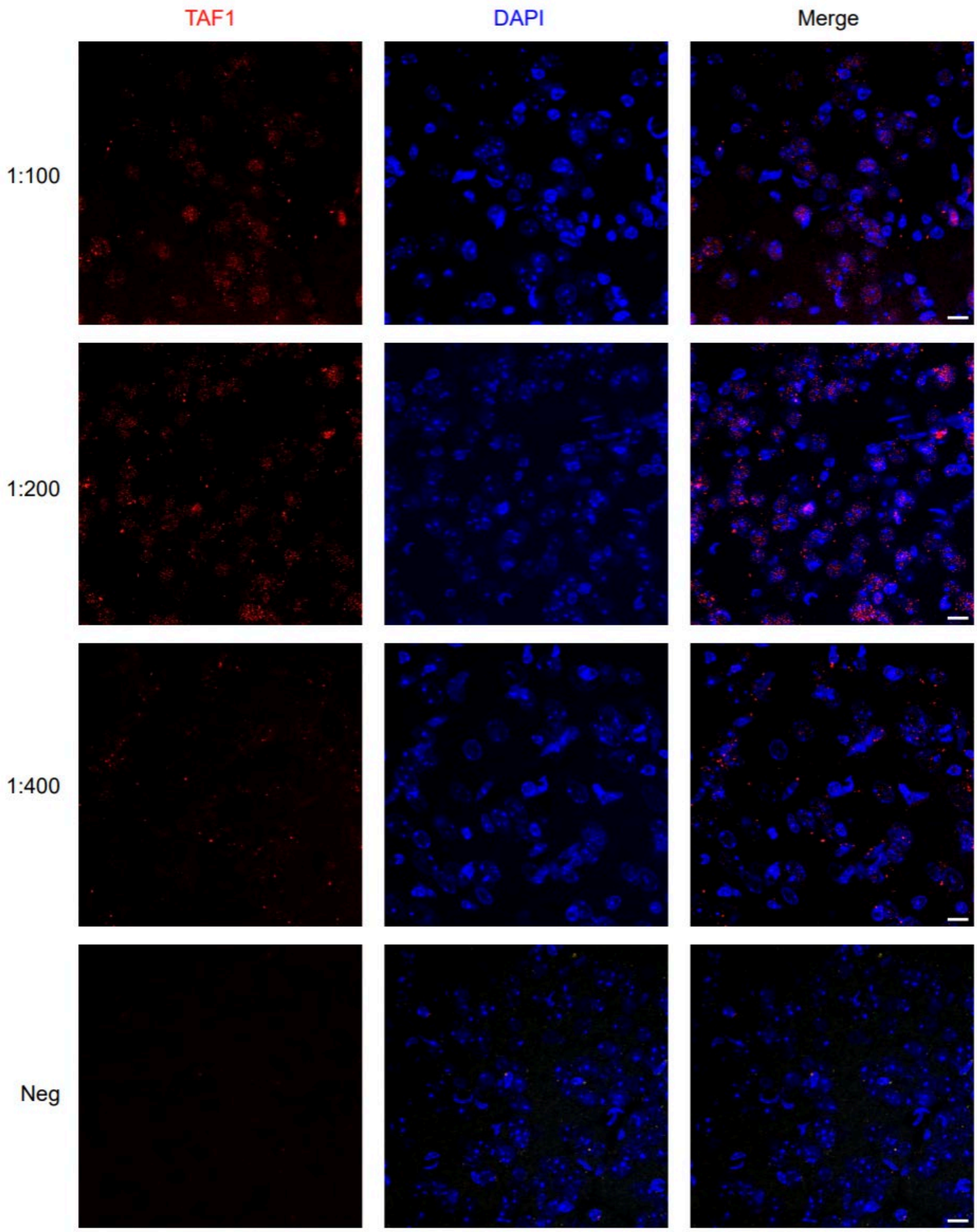

Fig. S1. Optimisation of dilutions for TAF1 antibody. TAF1 staining was optimised in the striatum with serial dilutions of primary antibody (1:100, 1:200, 1:400). The same concentration of secondary antibody (1:400), DAPI, and exposure for imaging was used for all sections. The negative control section (neg) was stained with DAPI and secondary antibody, with omission of only primary antibody. One section per antibody condition was used (N=1), where all sections came from the same Wt mouse sample. The experiment was repeated twice with reproducible results. This was not a quantitative experiment.

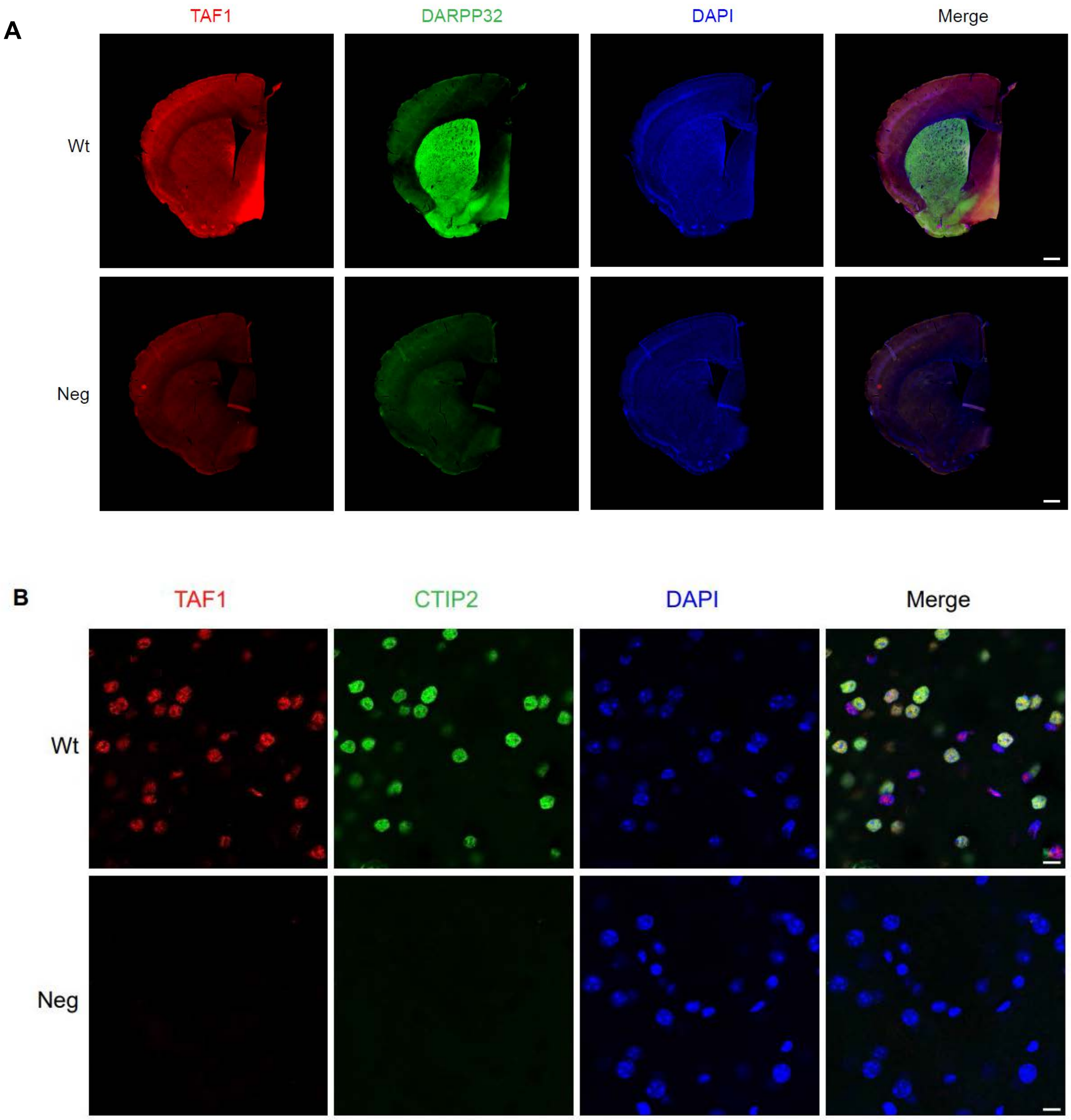

Fig. S2. TAF1 expression in mouse brain with negative controls. Immunostaining is shown for female mice of 3-5 months old, for wild-type (Wt) mice. (**A**) TAF1 (red) is co- stained with DARPP32 (green) which indicates medium spiny neurons (MSNs) in the triatum, and DAPI (blue) was used to stain all nuclei. Scale bar, 500 µm. (**B**) CTIP2 antibody (green) was used to co-stain medium spiny neuron (MSN) nuclei and DAPI (blue) was used to stain all nuclei. Negative control sections (neg) were stained with DAPI and secondary antibodies, with omission of only primary antibodies. One section per antibody condition was used (N=1), where all sections came from the same Wt mouse sample. The experiment was repeated twice with reproducible results. This was not a quantitative experiment.

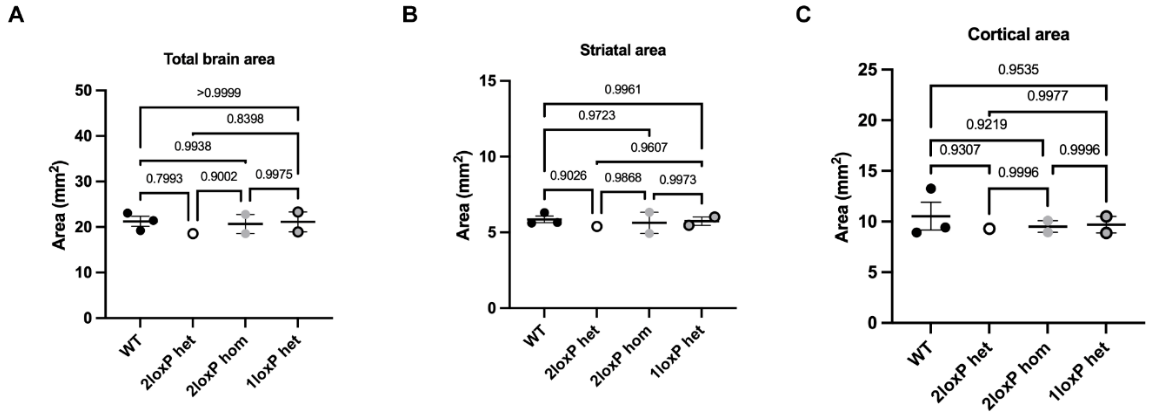

Fig. S3. Quantification of brain size from coronal sections. From serial sections of those in Figure 6, (**A**) total brain area, (**B**) striatal area, and (**C**) cortical area, were measured using H&E staining to identify brain regions. Each dot represents one replicate section from the same mouse (Wt, N=3; 2loxP het, N=1; 2loxP hom, N=2; 1loxP het, N=2). The experiment was not repeated. All groups were compared by one-way ANOVA, where Tukey's multiple comparison test was used to compare groups. Error bars show mean ± SEM, and P-values are indicated.

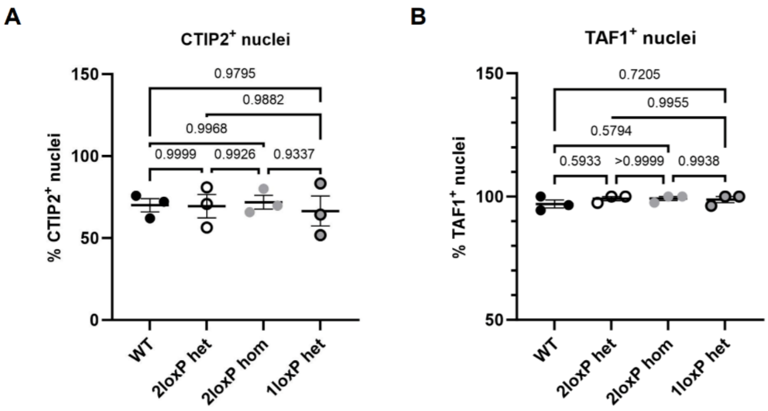

Fig. S4. Quantification of TAF1 expression in nuclei in striatum. From Figure 7, number of (**A**) CTIP2-positive and (**B**) TAF1-positive nuclei were counted, expressed as a percentage of the total nuclei labelled by DAPI staining. Each dot represents one sample from one mouse (n=3 mice/genotype). The experiment was not replicated. All groups were compared by one-way ANOVA, where Tukey's multiple comparison test was used to compare groups. Error bars show mean ± SEM, and P-values are indicated.

Table S1. Guides and templates for CRISPR reactions

Method	Nucleic Acid	Name	Sequence
Cytoplasmic injection	RNA guide	cgEMS23	CTCCCTAGTGCTGAGAACAG
	RNA guide	cgEMS30	GCGCCACCACGCCAGGCATG
	DNA template	oEMS6395	GTCTCTCTGTAGCACTGGCTGTCCCAGAATTCATTC<br>TATAGGTTAGGCTGACCTCAAATTCATAGAGATCAG<br>CCTGCCTCTGCCTCCCTAGTGCTGAGAAATAACTTC<br>GTATAGCATACATTATACGAAGTTATCAGAGGCAAG<br>AACCACCACTGCCCAGCTAAGCACAGTATTTTTTAT<br>TTTAACCTGGTTGAGAAAAAATTATAACATGATAAA<br>TATAAAGTGCCTTTATGAACAAAAAAATGAAAAAAA<br>AATCAGAGATTAGATTTGAAGCATTTCACTTTCTCT<br>CAGAAGCTTAAAAGAGCACAAGTAGGATTACTTATA<br>ATGACGGAGATAAAAGCCTGGATGTTAGATATTCCT<br>GGCATAGAAGCACACCTTGCTGCACATTGTAGGCCA<br>TGGCATTCCTAGTCATACTGGAAGGAAGCCAGCCTG<br>CCAAACTCGCACGCTGAGGTTTTGTTCCTTTGTGTT<br>TGACATCCTCCCCATCCCAAATGATATCATCTTCCC<br>AATGCAGCTGTGTCACCATTAGGAAGTTTTCATCTG<br>CCAGAAGATCGACGCCACTGTTTTCCTCAAGTTTCC<br>TTAATTTCTGTGTAAGTAGAAAGAAGAATCAACCAG<br>TCAGTCACTAATCAAAGGTCCCAATGCAGATCCTAA<br>ATGACTCAGGAAGTGGTGCTTTTCCAGGCTTAGTGT<br>CAAATACCTGCAGTGCTAACACTTGCGAGACTGAAA<br>GGAGAATTCCAAGTCTGAAGTCAGCCCAGGATACAA<br>AGTGTGACTCTGTTTCAAAAGCCAAGGATTGGCAAT<br>GTAGCTCAGTGATAAGAGCTTCCCCATATAACTTCG<br>TATAGCATACATTATACGAAGTTATGCCTGGCGTGG<br>TGGCGCACGCCTTTAATCCCAGCACTCGGGAGGCAG<br>AGGCAGGAGGATTTCTGAGTTTAAGGCCAGCCTAGT<br>CTACAAAGTGAGTTCC
iGONAD electroporation	RNA guide	cgEMS39	TTCACCAACTCTGAGGTCAG
	DNA template	oEMS6414	AGGGAAATCTTTTCTCAAAAATCCAATAAATCAAGG<br>TGACTTTTAAATATGCCCCCTCTGATAACTTCGTAT<br>AGCATACATTATACGAAGTTATACCTCAGAGTTGGT<br>GAAAGGGTTATCAAGATCACCAAGAATAATTTAGAG<br>GTTGCGGGGC

Disease Models & Mechanisms • Supplementary information

Table S2. Primers used for genotyping mice

Target	Name	Forward primer	Name	Reverse primer	Amplicon size
Taf1 2loxP (5' loxP size difference)	oEMS 6445	TGACTCCTAG GTCTTGCACA	oEMS 6446	AGGAAGTGGT GCTTTTCCAG	354 bp (no loxP) 388 bp (with loxP)
Taf1 2loxP (5' loxP specific)	oEMS 6443	CACGCCAGGCAT AACTTCGTATAAT	oEMS 6444	GCTTTGTTTG GCTAGTTGGTT	896 bp
Taf1 2loxP (3' loxP size difference)	oEMS 6447	CAGGCTTCCTA AGTACTACAAT	oEMS 6448	CTCAAAAATCCAA TAAATCAAGGTGAC	362 bp (no loxP) 396 bp (with loxP)
Taf1 2loxP (3' loxP specific)	oEMS 6447	CAGGCTTCCTA AGTACTACAAT	oEMS 6449	TATGCCCCCT CTGATAACTTC	362 bp
Taf1 1loxP (recomb.)	oEMS 6476	TAGGTCTTGC ACATGTTGGG	oEMS 6477	AGGCAAGACT GAAACATGCA	361 bp (with recomb.) 1,885 bp (no recomb.)
Cre (JAX Protocol)	oIMR 1084	GCGGTCTGGCA GTAAAAACTATC	oIMR 1085	GTGAAACAGCA TTGCTGTCACTT	100 bp
Sex (PMID 15945368)	Forward *Kdm5c*	CTGAAGCTTT TGGCTTTGAG	Revers e *Kdm5c*	CCACTGCCA AATTCTTTGG	302 bp (Y Chrom.) 331 bp (X Chrom.)

JAX, The Jackson Laboratory, Bar Harbor, ME; PMID, Pubmed Identifier

Disease Models & Mechanisms • Supplementary information

Table S3. Primers used for Samplix sequencing

Target	Name	Forward primer	Name	Reverse primer	Assay type
Upstream of 5' loxP	WO100017_Up_1F	ACTTTTGTGGTCTGAACTTAGCAC	WO100017_Up_1R	GAACATTCTTCCCCGGTCCA	dPCR main
Upstream of 5' loxP	WO100017_Up_3F	AACTTGTCCTGTTGGTGGCT	WO100017_Up_3R	CCAATTTGTGAGATCTGCAGAGC	qPCR main
Upstream of 5' loxP	WO100017_Dn_5F	ATGGACGTTGGGAGGACAAT	WO100017_Dn_5R	CTCCTGCCATCAACATACCCA	dPCR backup
Upstream of 5' loxP	WO100017_Dn_6F	AGCTGCCAGTTTTTAGTGTTTG	WO100017_Dn_6R	CACTACTGCCCAAGCCATGT	qPCR backup

dPCR, fluorescent in-droplet PCR; main, assay used in all cases; qPCR, quantitative PCR; backup, assay used in cases of DNA degradation

Table S4. Primers used for quantitative RT-PCR.

Target	Forward primer	Reverse primer
Taf1 Exon 7	CACAATGATGGCTCCTGTGGA	AAGCCACTGCCATCTTCAGG
Taf1 Exon 8	AGGAAAACAGTGGCGTCGAT	TGACATCCTCCCCATCCCAA
Taf1 Exon 34	AGTCTGGACCCAATGACTCC	AGCATCTCGAGAAACACTGAGG
Taf1 Exon 34’	GGCTAAGCCTCCTGATTTGT	CAAGACAGACAGATTGCTCTCA
Taf1 Exon 38	CCAGTTGGCACTTGAGACTCA	GGGTGGGAGGGAGATGAAGA
β-actin (*Actb*)	AGAAAATCTGGCACCACACC	AGAGGCGTACAGGGATAGCA

Table S5. Antibodies used for immunofluorescent staining (IF) and western blot (WB). Dilutions used are described in the materials and methods section.

Category	Target [clone]	Company	Catalogue no.	Batch no.	Application
Primary	TAF1 [177-4] * (rabbit monoclonal)	Timmers Lab supported by CCXDP (Capponi *et al.* (2020))	N/A (not commercially available)	N/A	IF/WB
Primary	CTIP2 [25B6] (rat monoclonal)	Abcam	ab18465	2101032938	IF
Primary	DARPP-32 [H-3] (mouse monoclonal)	Santa Cruz Biotechnology	sc-271111	1021	IF
Secondary	Anti-rat IgG (H+L) Alexa Fluor 488	Thermo Fisher	A-11006	2551392	IF
Secondary	Anti-mouse IgG (H+L) Alexa Fluor 488	Thermo Fisher	A-11001	2551357	IF
Secondary	Anti-rabbit IgG (H+L) Alexa Fluor 564	Thermo Fisher	A-11035	2387450	IF
Primary	β-tubulin [EPR16774] (rabbit monoclonal)	Abcam	ab179513	GR3214136-5	WB
Primary	α-actinin [H-2] (mouse monoclonal)	Santa Cruz	sc-17829	L2921	WB
Secondary	Anti-rabbit IgG (H+L) IRDye 800CW	LI-COR	926-32211	D10512-05	WB
Secondary	Anti-mouse IgG (H+L) IRDye 680LT	LI-COR	926-68020	D11005-15	WB

* Validation of this TAF1-specific monoclonal antibody has been published in Capponi et al. (2020). The epitope is TPGPYTPQPPDLY derived from peptides encoded by exons 34 and 35 of mouse *Taf1 (Taf1*-201 *and Taf1*-202).
